# JNK signaling coordinates epithelial cell turnover through exocytosis in *Drosophila* ribosomal protein mutants

**DOI:** 10.1016/j.isci.2025.112587

**Published:** 2025-05-05

**Authors:** Nanami Akai, Yoshimasa Yagi, Tatsushi Igaki, Shizue Ohsawa

**Affiliations:** 1 Group of Genetics, Division of Biological Science, Graduate School of Science, Nagoya University, Furo-cho, Chikusa-ku, Nagoya, Aichi 464-8602, Japan; 2 Laboratory of Genetics, Graduate School of Biostudies, Kyoto University, Yoshida-Konoe-cho, Sakyo-ku, Kyoto 606-8501, Japan

**Keywords:** Cell biology, Functional aspects of cell biology, Developmental biology

## Abstract

Robust tissue growth is orchestrated by the precise coordination of cell death and cell proliferation. In the developing wing pouches of *Drosophila Minute*/+ animals, both cell death and compensatory cell proliferation are increased, thereby contributing to robust growth of mutant tissue. Here, we show that JNK-mediated elevation of exocytosis in dying cells is crucial for triggering cell turnover in *M/+* wing morphogenesis. Mechanistically, elevated JNK signaling in dying cells upregulates exocytosis-related genes and Wingless (Wg), leading to enhanced Wg secretion. Notably, increased exocytosis promotes caspase activation via an exocytosis-Dronc amplification loop, sustaining apoptotic signaling while reinforcing Wg secretion through Dronc activation. Furthermore, this exocytosis-mediated Wg secretion and apoptotic feedback loop universally occurs downstream of JNK signaling, regardless of the genetic background. Overall, our findings provide mechanistic insights into robust tissue growth through the orchestration of cell turnover, primarily via JNK-mediated exocytosis during *Drosophila Minute/+* wing morphogenesis.

## Highlights

• JNK-exocytosis axis coordinates robust tissue growth in *Minute/+* wing morphogenesis
• JNK signaling upregulates exocytosis-related genes and Wg, leading to Wg secretion
• Elevated exocytosis promotes caspase activation via a feedback loop with Dronc

## Graphical abstract

**Figure:**
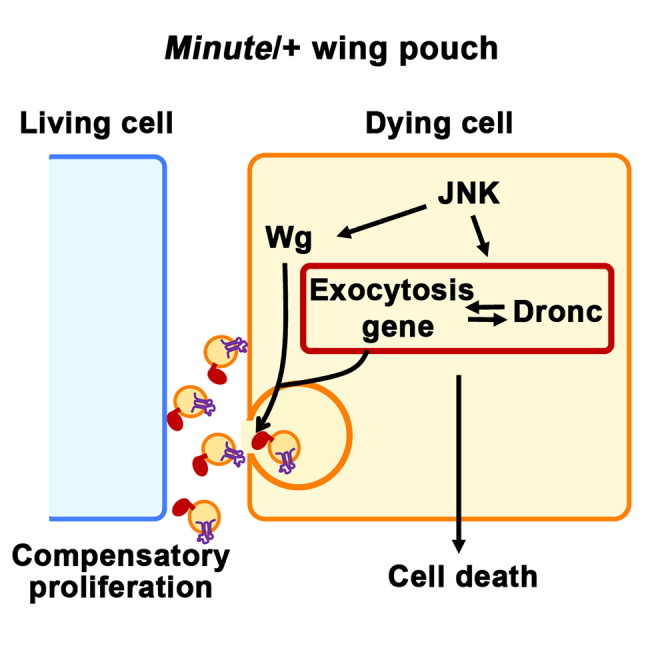

## Introduction

Ribosomes are essential molecular machines responsible for protein synthesis in living cells, and thus fundamental to life. In humans, heterozygosity of genes related to ribosomal proteins or ribosomal biogenesis factors leads to a group of genetic disorders collectively termed ribosomopathies. For instance, heterozygous mutations in various ribosomal protein genes, including *RPS17*,^1^ *RPS26*,^2^ *RPS19*,^3^ *RPS24*,^4^ and *RPL27*,^5^ are associated with Diamond-Blackfan anemia (DBA), with some patients exhibiting tissue-specific developmental anomalies, such as limb defects, cleft palate, and abnormal heart development.^6^^,^^7^ In addition, heterozygous mutations in the *RpSA* gene are linked to isolated congenital asplenia (ICA), a disorder characterized by the absence of a spleen at birth.^8^ However, the precise mechanisms by which a reduction in ribosomal protein gene dosage leads to these genetic disorders remain elusive.

In *Drosophila*, a series of heterozygous mutants for ribosomal protein genes, called *Minute/+* (*M/+*), exhibit a pronounced developmental delay during larval stages.^9^ Despite this significant delay, *M*/+ animals are essentially normal flies without noticeable morphological defects, except for the thinner bristles, suggesting that *M/+* animals exert certain mechanisms to overcome developmental perturbations caused by reduced ribosomal protein levels. It has been reported that extensive cell death,^10^ as well as cellular stress,^10^^,^^11^^,^^12^^,^^13^ is a distinctive characteristic of the *M*/+ wing pouch. Our previous study^14^ has shown that apoptotic cell death and the subsequent cell proliferation are dramatically increased in the *M*/+ wing pouch. Blocking this cell turnover by inhibiting cell death resulted in morphological abnormalities, indicating the essential role of cell turnover in *M/+* wing morphogenesis. Genetic analyses have revealed that the induction of this cell turnover depends on activation of JNK (c-Jun N-terminal kinase) signaling. However, downstream events of JNK activation have remained to be elucidated.

The coordination of cell death and proliferation through cell-cell communications is crucial for proper development and homeostasis of multicellular organisms. Apoptotic cells, for example, can secrete mitogens such as Wingless (Wg; a Wnt homolog), dpp (a BMP homolog), and Hh, which could promote the proliferation of nearby cells in the *Drosophila* epithelium.^15^^,^^16^^,^^17^^,^^18^^,^^19^ Cell competition, a phenomenon in which cells with higher fitness (“winners”) eliminate neighboring less fit cells (“losers”) by inducing cell death,^20^ is another aspect of cell turnover. It has been reported that secretory factors from prospective loser cells contribute to cell competition. For instance, the *Drosophila* cytokine Unpaired 3 (Upd3), which is produced by losers, promotes cell competition between wild-type (winners) and *M/+* cells (losers) in the epithelium.^12^ Furthermore, Madin-Darby canine kidney cells depleted of the polarity regulator gene *scribble* (*scrib*^KD^ MDCK cells) secrete fibroblast growth factor 21 (FGF21), which in turn promotes cell competition with neighboring wild-type MDCK cells.^21^ However, the mechanism by which these secreted factors are released from dying cells is still not fully understood.

Here, we found that exocytosis, acting as a downstream event of JNK signaling in dying cells, contributes to cell turnover in the *M/+* wing pouch, which is essential for robust wing development in *M*/+ animals. Our genetic analyses suggest that increased exocytosis forms a signal amplification loop with Dronc, sustaining apoptotic signaling and reinforcing the release of Wg from dying cells, which in turn stimulates their own cell death and the proliferation of neighboring living cells. Furthermore, we found that this exocytosis-mediated Wg release is not restricted to *M*/+ context but appears to represent a universal event downstream of JNK signaling. Our findings provide new mechanistic insights into how dying cells coordinate robust development through cell-cell interactions.

## Results

### *M/+* wing pouch elevates exocytosis downstream of JNK signaling

To elucidate the downstream event of JNK signaling in the *M/+* wing pouch, we conducted RNA sequence (RNA-seq) analyses on GFP-labeled fluorescence-activated cell sorting (FACS)-sorted wing pouch cells from one of the *M/+* mutants *RpS3/+*, compared to GFP-labeled wild-type control or *RpS3/+* wing pouch cells overexpressing JNK inhibitor Puckered (Puc) (Figure S1A; the wing pouch is green-marked oval domain that becomes the adult wing blade). Among the 1,097 genes that were upregulated or downregulated in the *RpS3/+* wing pouch relative to the wild-type control, we identified JNK targets or positive regulators including *reaper*^22^ and *Gadd45*,^23^ JAK/STAT targets including *Socs36E*^24^ and c*hinmo*,^25^ Gr64 gustatory receptors and numerous genes associated with oxidative stress and DNA repair (Figure S1B; Tables S1 and S2), which aligns with previous reports on genes upregulated in the *RpS3/+* or *RpS17/+* wing disc.^12^^,^^13^ These data validate our experimental conditions for identifying genes required for JNK-mediated events in the *M/+* wing pouch.

Mining the list of genes differentially expressed in the *RpS3/+* wing pouch cells dependent on JNK signaling (Figure S1C; Table S3), we noticed that among the genes associated with the “secretion by cell” GO term (Figure S1B), the evolutionarily conserved exocytosis-related genes *unc-13*, *SNAP25*, and *Cadps* (*calcium-dependent secretion activator*) were upregulated in a JNK-dependent manner (2.29-fold increase, *RpS3*/+ compared to wild-type; 3.44-fold increase, *RpS3*/+ compared to *RpS3*/+ + Puc) (Figure S1D). Among these exocytosis-related genes, the upregulation of *SNAP25* and *unc-13* was consistent with the previous reports concerning genes upregulated in *RpS3/+* or *RpS17/+* cells.^12^^,^^13^

**Figure 1:**
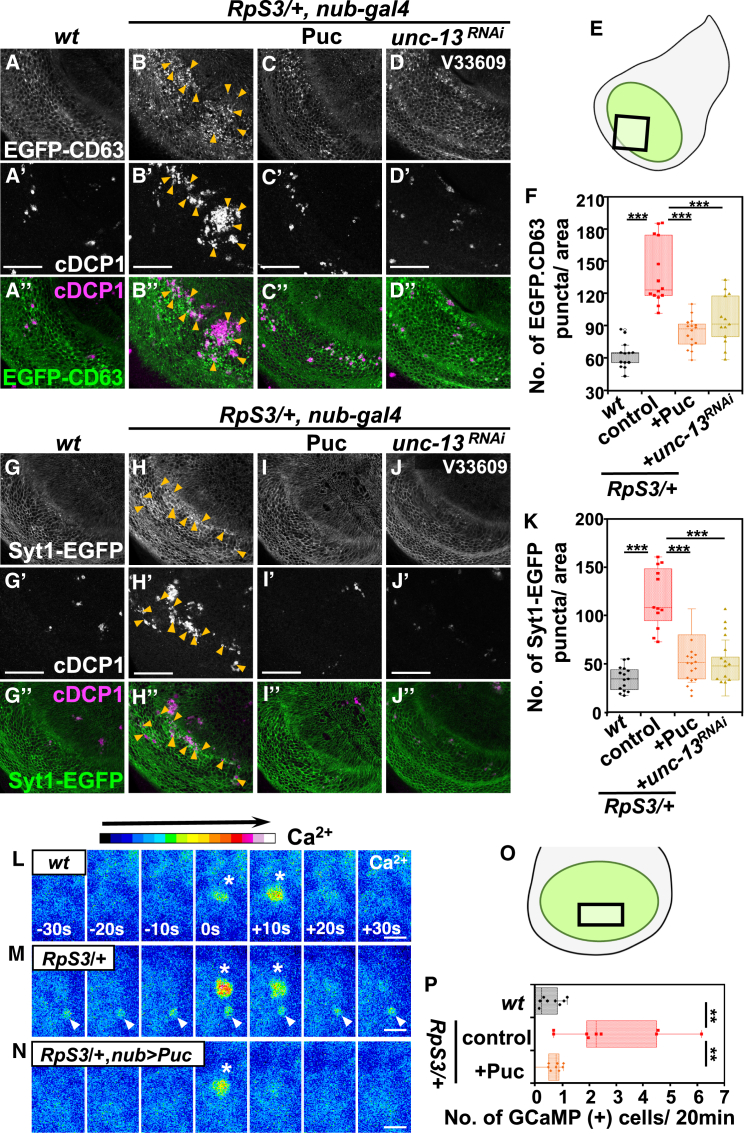
*M/+* wing pouch elevates exocytosis downstream of JNK signaling (A–D’’) The exosome marker EGFP-CD63 was expressed in the wing pouch of wild-type (A), *RpS3/+* (B), *RpS3/+*, *nub-Gal4*, *UAS-Puc* (C), or *RpS3/+*, *nub-Gal4*, *UAS-unc-13-RNAi* (D) flies (white). The images correspond to the boxed area in the schematic diagram of the wing disc (E). Dying cells in the wing disc were visualized by anti-cleaved Dcp-1 staining (white). EGFP-CD63-positive vesicles were frequently observed in the area of morphological dying cells in the *RpS3/+* wing pouch (Indicated by Orange arrowheads). Scale bar, 50 μm. (F) Boxplot with dots representing the number of EGFP-CD63-positive puncta in the pouch of genotypes shown in (A) (*n* = 13, number of wing pouches), (B) (*n* = 14), (C) (*n* = 16), and (D) (*n* = 13). Thick line, median; ∗∗∗, *p* < 0.001; Wilcoxon rank-sum test. (G–J’’) The vesicle marker Syt1-EGFP was expressed in the wing pouch of wild-type (G), *RpS3/+* (H), *RpS3/+*, *nub-Gal4*, *UAS-Puc* (I), or *RpS3/+*, *nub-Gal4*, *UAS-unc-13-RNAi* (J) flies (white). The images correspond to the boxed area in the schematic diagram of the wing disc (E). Dying cells in the wing disc were visualized by anti-cleaved Dcp-1 staining (white). Syt1-EGFP-positive vesicles were frequently observed in the area of morphological dying cells in the *RpS3/+* wing pouch (Indicated by Orange arrowheads). Scale bar, 50 μm. (K) Boxplot with dots representing the number of Syt1-EGFP-positive puncta in the wing pouch of genotypes shown in (G) (*n* = 15, number of wing pouches), (H) (*n* = 12), (I) (*n* = 14), and (J) (*n* = 16). Thick line, median; ∗∗∗*p* < 0.001; Wilcoxon rank-sum test. (L–N) Time-lapse imaging of Ca^2+^ signaling in cultured wing discs. The calcium reporter GCaMP6m (shown in pseudo color) was expressed in the wing pouch of wild-type (L), *RpS3/+* (M), or *RpS3/+*, *nub-Gal4*, *UAS-Puc* (N) flies. Images were acquired at 10-s intervals. White arrowheads indicate the persistent activation of Ca^2+^ signaling. White asterisks indicate spontaneous Ca^2+^ flashes. The images correspond to the boxed area in the schematic diagram of the wing disc (O). Scale bar, 5 μm. (P) Quantification of cells exhibiting continuous Ca^2+^ activity for 20 min in the wing pouch. The respective genotypes are shown in (L) (*n* = 12), (M) (*n* = 10), and (N) (*n* = 9). These images are Z-stacked images from 3 confocal images (4 μm). Thick line, median; ∗∗*p* < 0.01; Wilcoxon rank-sum test.

**Figure 2:**
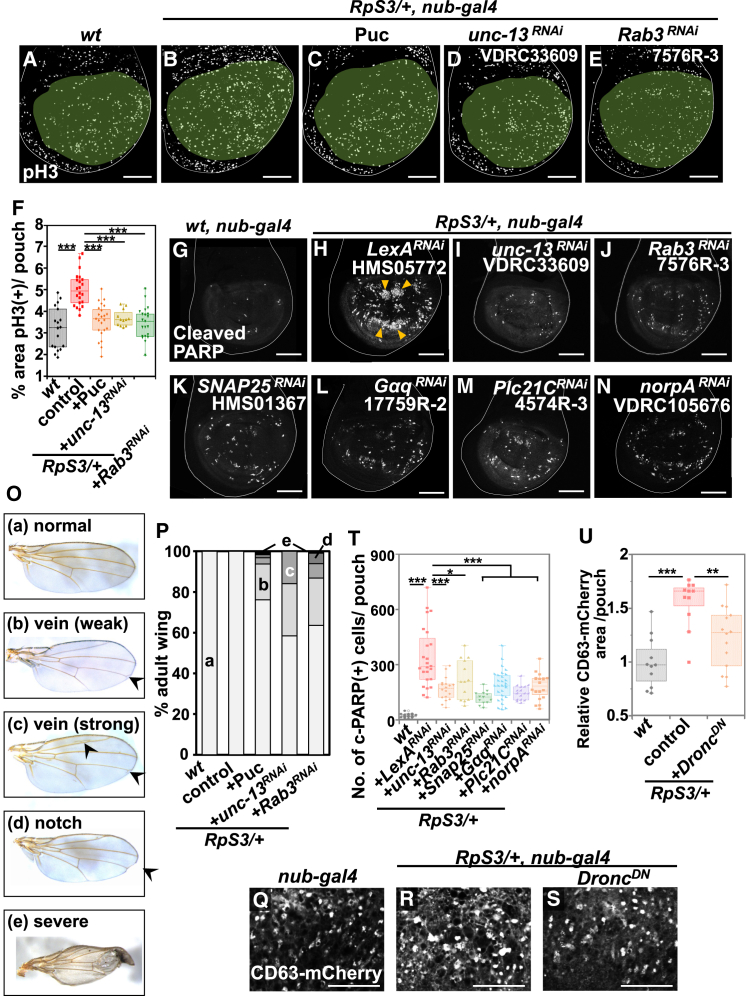
Exocytosis is required for massive cell turnover in the *M/+* wing pouch (A–E) Wing discs of wild-type (A), *RpS3/+* (B), *RpS3/+*, *nub-Gal4*, *UAS-Puc* (C), *RpS3/+*, *nub-Gal4*, *UAS-unc-13-RNAi* (D), or *RpS3/+*, *nub-Gal4*, *UAS-Rab3-RNAi* (E) flies were stained with anti-phospho-histone H3 (pH3) (Ser10) antibody (white). Wing pouches were marked in pale green. Scale bar, 100 μm. (F) Boxplot with individual dots representing pH3-positive areas in the pouch of genotypes shown in (A) (*n* = 18, number of wing pouches), (B) (*n* = 22), (C) (*n* = 23), (D) (*n* = 12), and (E) (*n* = 21). Thick line, median; ∗∗∗*p* < 0.001; Wilcoxon rank-sum test. (G–N) The activated-caspase-3 indicator CD8-PARP-Venus was expressed in the wing pouch of wild-type (G), *RpS3/+*, *nub-Gal4*, *UAS-lexA-RNAi* (H), *RpS3/+*, *nub-Gal4*, *UAS-unc-13-RNAi* (I), *RpS3/+*, *nub-Gal4*, *UAS-Rab3-RNAi* (J), *RpS3/+*, *nub-Gal4*, *UAS-SNAP25-RNAi* (K), *RpS3/+*, *nub-Gal4*, *UAS- Gaq-RNAi* (L), *RpS3/+*, *nub-Gal4*, *UAS-Plc21C-RNAi* (M), or *RpS3/+*, *nub-Gal4*, *UAS-norpA-RNAi* (N) flies, and dying cells in the wing pouch were visualized by anti-cleaved PARP staining (white). Orange arrowheads indicate massive cell death in the *RpS3/+* wing pouch. Scale bar, 100 μm. (O) Adult wing phenotypes were classified as following five types: (a) normal, (b) vein (weak) (weakly bearing a brunched vein in the point of “landmark 14” (arrow)^41^ (c) vein (strong) (bearing other additional vein phenotypes (arrowheads) in addition to “landmark 14”), (d) notch, and (e) severe. (P) The rate of defective wings in the genotypes of wild-type (*n* = 55, number of adult wings), *RpS3/*+ (*n* = 44), *RpS3/+*, *nub-Gal4*, *UAS-Puc* (*n* = 63), *RpS3/+*, *nub-Gal4*, *UAS-unc-13-RNAi* (*n* = 101), and *RpS3/+*, *nub-Gal4*, *UAS-Rab3-RNAi* (*n* = 99). (Q–S) The exosome marker CD63-mCherry was expressed in the wing pouch of wild-type (Q), *RpS3/+* (R), or *RpS3/+*, *nub-Gal4*, *UAS-Dronc*^*DN*^ (S) flies (white). The images correspond to the area enclosed by rectangle 1 in the schematic diagram of the wing disc (Figure 3C). Scale bar, 20 μm. (T) Boxplot with individual dots representing the number of cleaved-PARP-positive dying cells per pouch in genotypes shown in (G) (*n* = 18, number of wing pouches), (H) (*n* = 24), (I) (*n* = 18), (J) (*n* = 13), (K) (*n* = 13), (L) (*n* = 29), (M) (*n* = 16), and (N) (*n* = 19). Thick line, median; ∗∗*p* < 0.001, ∗*p* < 0.05; Wilcoxon rank-sum test. (U) Boxplot with dots representing the number of CD63-mCherry-positive puncta in the pouch of genotypes shown in (Q) (*n* = 11, number of wing pouches), (R) (*n* = 11), and (S) (*n* = 14). Thick line, median; ∗∗∗*p* < 0.001, ∗∗*p* < 0.01; Wilcoxon rank-sum test.

Calcium-dependent exocytosis is the process whereby cells release intracellular contents into the extracellular space. It has been shown that *unc-13*, *SNAP25*, and *cadps* collectively regulate the docking process of secretory vesicles to the plasma membrane during Ca^2+^-mediated exocytosis.^26^ Specifically, UNC-13, a conserved presynaptic protein with calcium-binding domains, interacts with syntaxin to prime vesicles for fusion, crucial for calcium-regulated exocytosis.^27^ SNAP-25, in conjunction with syntaxin-1 and synaptobrevin, forms the pivotal SNARE complex for neuronal exocytosis, assembling into a four-helix bundle that is essential for drawing vesicle and plasma membranes close together to enable membrane fusion.^28^^,^^29^ Like UNC-13, CAPS/Cadps possesses conserved C-terminal domains that are instrumental in the assembly of SNARE complexes, thus priming vesicles for Ca^2+^-induced exocytosis.^30^ First, to investigate whether exocytosis is upregulated in the *M/+* wing pouch, we utilized the two exosome markers, CD63 tagged with EGFP and mCherry (EGFP-CD63 and CD63-mCherry).^31^^,^^32^^,^^33^^,^^34^^,^^35^^,^^36^^,^^37^ In addition, we utilized Syt1 tagged with EGFP (Syt1-EGFP), a widely used vesicle marker prevalent in both neuronal and non-neuronal cells.^38^^,^^39^^,^^40^ We observed a significant increase in EGFP-CD63-positive and Syt1-EGFP-positive vesicles in the *RpS3/+* wing pouch compared to the wild-type control (Figures 1A, 1B, 1G, and 1H, corresponding to the boxed region in Figure 1E, quantified in Figures 1F and 1K). EGFP-CD63-positive vesicles were also found to be increased in the *RpL19/+* wing pouch (Figure S1E, quantified in Figure S1F). Notably, these vesicles were predominantly observed in regions of the *RpS3/+* wing pouch undergoing massive cell death (as indicated by arrowheads in Figures 1B, 1H, and S1E), a phenomenon we previously reported^14^ and also confirmed in this study (shown in Figure 2H, compared to Figure 2G, quantified in Figure 2T). Furthermore, we observed a significant increase in extracellular vesicles, including CD63-mCherry-positive and Syt1-EGFP-positive vesicles, in the *RpS3/+* wing pouch compared to the wild-type control, as detected by staining with anti-mCherry or anti-GFP antibodies under non-permeabilizing conditions (Figures S1G, S1H, S3M, and S3N, quantified in Figure S1I and S3O).

To further investigate the enhanced exocytosis in the *RpS3/+* wing pouch, we utilized the GCaMP6m fluorescent calcium reporter^42^ to monitor Ca^2+^ activity, a known inducer of exocytosis. Persistent Ca^2+^ activity was observed in the area of massive cell death within the *RpS3/+* wing pouch, along with spontaneous Ca^2+^ flashes similar to those present in wild-type control cells (Figures 1L and 1M, quantified in Figure 1P). Notably, these cells exhibiting persistent Ca^2+^ activity in the *RpS3/+* wing pouch were SYTOX-positive, a dye that fluoresces upon binding DNA in cells with compromised plasma membranes, a characteristic of non-viable cells^43^ (Figure S1J). These observations suggest a potential link between persistent Ca^2+^ activity and an enhanced exocytic process in dying cells, potentially driven by elevated calcium levels in the *RpS3/+* wing pouch.

We then examined whether exocytosis is the downstream event of JNK activation in the *M/+* wing pouch. We found that blocking JNK signaling by overexpressing Puc significantly suppressed the increased number of EGFP-CD63/Syt1-EGFP-positive vesicles, as well as the emergence of cells exhibiting persistent Ca^2+^ signaling (Figure 1C, quantified in Figure 1F; Figure 1I, quantified in Figure 1K; Figure 1N, quantified in Figure 1P). Together, these observations suggest that exocytosis is elevated in dying cells within the *M/+* wing pouch as a downstream event of JNK signaling.

### Exocytosis is required for massive cell turnover in the *M/+* wing pouch

We have previously demonstrated that JNK signaling is required for cell turnover in the *M/+* wing pouch.^14^ Blocking JNK signaling by overexpressing Puc significantly reduced cell death and mitoses in the *RpS3*/+ wing pouch, as assayed by the CD8-PARP-Venus probe for caspase activity in the imaginal disc,^44^^,^^45^^,^^46^^,^^47^^,^^48^^,^^49^^,^^50^^,^^51^ and the M phase marker phospho-histone H3 (Figures 2A–2C, quantified in Figure 2F; Figures 2G, 2H, and S2A, quantified in Figure S2Q). In addition, we intriguingly found that downregulating *unc-13*, a docking factor involved in exocytosis, similarly diminished cell death and mitoses (Figures 2I and S2B, quantified in Figures 2T and S2Q, respectively; Figure 2D, quantified in Figure 2F), and also reduced EGFP-CD63-positive and Syt1-EGFP-positive vesicles in the *RpS3*/+ wing pouch (Figures 1D and 1J, quantified in Figures 1F and 1K, respectively). Knocking down *rab3*, a “Secretory GTPase Rabs” required for vesicle exocytosis, also significantly reduced cell death and mitoses in the *RpS3*/+ wing pouch (Figures 2J and S2C, quantified in Figures 2T and S2Q, respectively; Figure 2E, quantified in Figure 2F). Furthermore, overexpressing Puc or downregulating either *unc-13* or *rab3* led to morphological defects in the *RpS3/+* adult wing, while their individual overexpression or downregulation did not affect wing development (Figures 2O, 2P, and S2R). These results suggest that exocytosis, as a downstream component of JNK signaling, is pivotal for cell turnover and subsequent normal morphogenesis in the *M/+* wing pouch. Indeed, downregulating other docking factors such as *SNAP25* or *Syt1*,^52^ another Secretory GTPase *Rab27*, or *ALIX*, an ESCRT (endosomal sorting complexes required for transport)-related protein involved in the biogenesis of extracellular vesicles,^53^^,^^54^ also significantly suppressed cell death in the *RpS3*/+ wing pouch (Figure 2K, quantified in Figure 2T; Figures S2D–S2J, quantified in Figure S2Q). Additionally, downregulating factors, such as *Gαq*, *Plc21C*, and *norpA*, which are required for intracellular Ca^2+^ release that triggers exocytosis process,^39^ also significantly suppressed cell death in the *RpS3*/+ wing pouch (Figures 2L–2N, quantified in Figure 2T; Figures S2K–S2P, quantified in Figure S2Q). Together, these data reinforce the vital role of exocytosis in cell turnover within the *M*/+ wing pouch, functioning as a downstream process of JNK signaling. Intriguingly, blocking Dronc activity by overexpressing Dronc^DN^ suppressed the elevation of exocytosis in the *RpS3*/+ wing pouch (Figure 2S, compared to Figure 2R, quantified in Figure 2U). This suggests the presence of a signal amplification loop between exocytosis and Dronc within the JNK signaling pathway, consistent with previous studies showing Dronc-JNK amplification loop that induce cell death.^55^^,^^56^

### Dying cells secrete Wg via exocytosis in the *M/+* wing pouch

We next sought to identify the protein secreted through exocytosis from dying cells in the *M/+* wing pouch. We found that *wg* expression was specifically upregulated in dying cells within the *RpS3/+* wing pouch, compared to the wild-type control (Figures S3A and S3B), as assayed by the *wg-lacZ* reporter.^57^ This elevated *wg* expression was significantly suppressed by overexpressing Puc in the *RpS3/+* pouch (Figure S3C, quantified in Figure S3D), suggesting that *wg* expression is mediated through JNK signaling in this context, which is consistent with the previous studies showing that apoptotic cells produce Wg through JNK signaling.^18^^,^^58^^,^^59^ Additionally, our previous study^14^ suggested that cell turnover in the *M/+* wing pouch is initiated by a mechanism akin to Wg-dependent cell competition, a phenomenon by which cells with higher Wg signaling activity eliminate neighboring cells with lower Wg signaling activity in the wing disc.^60^ Indeed, as demonstrated through previous genetic manipulations,^14^ and confirmed here, genetically reducing the Wg signaling gradient in the entire *M/+* pouch significantly suppressed cell death (Figure S3F, quantified in Figure S3G). These observations led us to hypothesize that dying cells in the *M/+* wing pouch might release Wg via JNK-mediated exocytosis, potentially conferring a survival advantage to their neighboring living cells. Supporting this hypothesis, it has been reported that Wnt/Wg can be released through extracellular vesicles including exosomes.^33^^,^^61^^,^^62^^,^^63^^,^^64^

**Figure 3:**
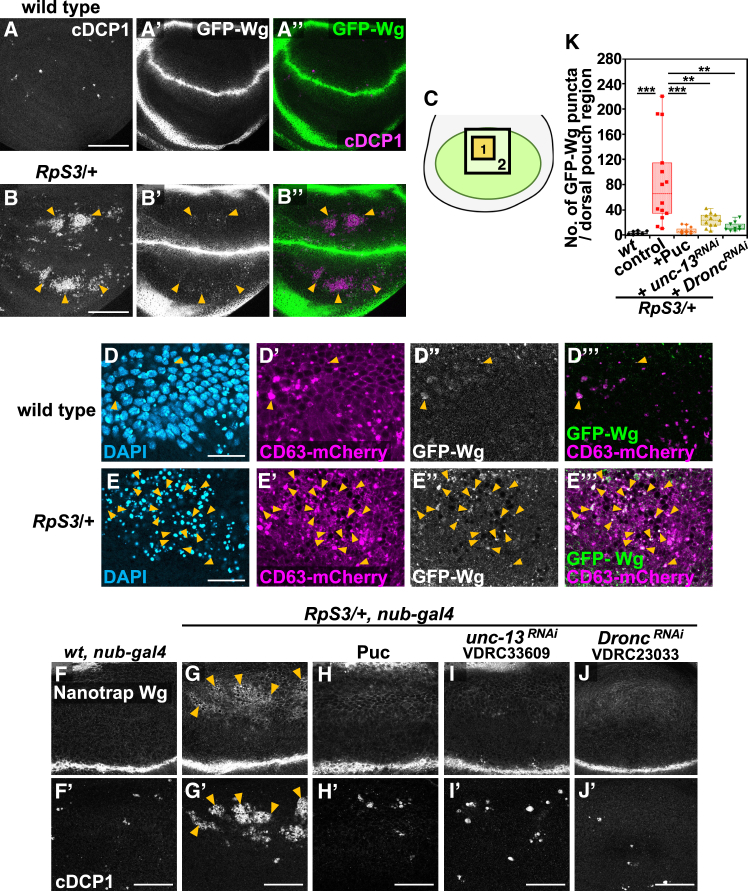
Dying cells secrete Wg via exocytosis in the *M/+* wing pouch (A and B’’) Wing discs of *GFP-Wg/*+ (A), or *RpS3/+*, *GFP-Wg/+* (B) flies, and dying cells in the wing pouch were visualized by anti-cleaved Dcp-1 staining (white). GFP-Wingless was visualized by anti-GFP staining (white). Arrowheads indicate massive cell death in the *RpS3/+* wing pouch. Scale bar, 100 μm. (D–E”’) Immunofluorescent localization of the exosome marker CD63-mCherry, as visualized by anti-DsRed staining (magenta), and GFP-Wingless (white) in the wing pouch of *GFP-Wg/+* (D), or *RpS3/+*, *GFP-Wg/+* (E) flies expressing CD63-mCherry. The nuclei were visualized by DAPI staining (blue). The images correspond to the area enclosed by rectangle 1 in the schematic diagram of the wing disc (C). Orange arrowheads indicate the colocalization of GFP-Wingless-positive puncta with CD63-mCherry-positive puncta. Scale bar, 20 μm. (F–J’) The membrane-tethered anti-GFP nanobody (Vhh4-CD8-HA) was expressed in the wing pouch of *GFP-Wg/+* (F), *RpS3/+*, *GFP-Wg/+* (G), *RpS3/+*, *GFP-Wg/+*, *nub-Gal4, UAS-Puc* (H), *RpS3/+*, *GFP-Wg/+*, *nub-Gal4*, *UAS-unc-13-RNAi* (I), or *RpS3/+*, *GFP-Wg/+*, *nub-Gal4*, *UAS-Dronc-RNAi* (J) flies. GFP-Wingless was visualized by anti-GFP staining (white). Dying cells were visualized by anti-cleaved Dcp-1 staining in the wing discs (white). The images correspond to the area enclosed by the rectangle 2 in the schematic diagram of the wing disc (C). Immobilized GFP-Wg-positive puncta were frequently observed in the area of morphological dying cells in the *RpS3/+* wing pouch (Indicated by orange arrowheads). Scale bar, 50 μm. (K) Boxplot with individual dots representing the number of GFP-wingless-positive puncta per rectangle-2 in the wing pouch (C). The respective genotypes are shown in (F) (*n* = 9, number of wing pouches), (G) (*n* = 14), (H) (*n* = 11), (I) (*n* = 11), and (J) (*n* = 9). Thick line, median; ∗∗∗*p* < 0.001, ∗∗*p* < 0.01; Wilcoxon rank-sum test.

Consistently, we found that GFP-Wg-positive puncta, derived from a knock-in allele,^65^ were more abundant in the *RpS3/+* wing pouch compared to the wild-type control (Figures 3A and 3B). This increase in GFP-Wg-positive puncta, particularly in the area with massive cell death within the *RpS3*/+ wing pouch (Figures S3I–S3J’’’, compared to Figure S3H), was more evident when using a membrane-tethered anti-GFP nanobody (Vhh4-CD8), which immobilizes GFP-Wg on the cell surface^65^ (Figures 3F and 3G, quantified in Figure 3K). Notably, these GFP-Wg-positive puncta, observed both without and with the Vhh4-CD8 morphotrap, sometimes colocalized with CD63-mCherry-positive vesicles (Figures 3E and S3L, indicated by arrowheads), suggesting that GFP-Wg might be secreted via extracellular vesicles through JNK-mediated exocytosis. Supporting this possibility, we found that blocking JNK signaling by overexpressing Puc or knocking down *unc-13* significantly reduced the number of GFP-Wg-positive puncta in the *RpS3/+* wing pouch (Figures 3H and 3I, quantified in Figure 3K). Additionally, consistent with the exocytosis-Dronc amplification loop, knocking down *Dronc* similarly reduced the number of GFP-Wg-positive puncta in the *RpS3/+* wing pouch (Figure 3J, quantified in Figure 3K), suggesting that caspase activation reinforces Wg-vesicle accumulation.

Furthermore, we observed a significant increase in extracellular GFP-positive puncta and extracellular CD63-mCherry-positive vesicles in the *RpS3/+* wing pouch when GFP-Wg was captured on the cell surface using the Vhh4-CD8 morphotrap, compared to the wild-type control (Figures S3M and S3N, quantified in Figures S3O and S3P). These extracellular GFP-Wg-positive puncta occasionally colocalized with extracellular CD63-mCherry-positive vesicles (6.88% in the wild-type, and 16.45% in the *RpS3/+* wing pouch region, characterized by massive cell death, as indicated by the arrowheads in Figures S3M and S3N). Together, these data suggest that dying cells secrete Wg via exocytosis in the *M/+* wing pouch.

### JNK signaling triggers exocytosis-mediated Wg secretion

Finally, we investigated whether JNK activation universally upregulates exocytosis and subsequent Wg secretion in contexts beyond the *M/+* wing pouch. To address this, we generated JNK-activating clones through the overexpression of Eiger, the *Drosophila* homolog of tumor necrosis factor (TNF), in the eye discs. Eiger has been shown to specifically activate the JNK pathway through dTAK1 (*Drosophila* JNKKK) and Hep (Hemipterous; *Drosophila* JNKK).^66^

**Figure 4:**
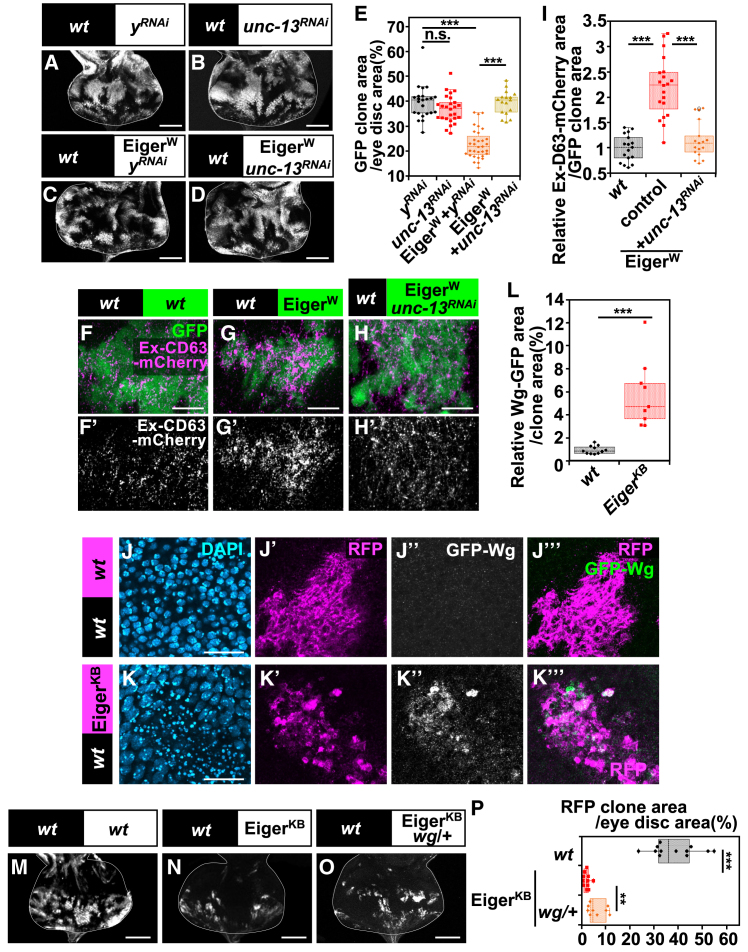
JNK signaling universally triggers exocytosis-mediated Wg secretion (A–D) Eye disc bearing eyFLP-induced MARCM clones of *UAS-yellow-RNAi* (A), *UAS-unc-13-RNAi* (B), *UAS-Eiger*^*W*^ +*UAS-yellow-RNAi* (C), or *UAS-Eiger*^*W*^*+UAS-unc-13-RNAi* (D) cells. Scale bar, 100 μm. (E) Boxplot with individual dots representing the clone size (% of total clone area per eye disc area) in genotypes shown in (A) (*n* = 23, number of eye discs), (B) (*n* = 26), (C) (*n* = 32), and (D) (*n* = 17). Thick line, median; ∗∗∗*p* < 0.001; n.s., not significant; Wilcoxon rank-sum test. (F–H) Eye disc bearing eyFLP-induced MARCM clones of *UAS-CD63-mCherry* (F), *UAS-Eiger*^*W*^ +*UAS-CD63-mCherry* (G), or *UAS-Eiger*^*W*^*+UAS-unc-13-RNAi* +*UAS-CD63-mCherry* (H) cells, stained with anti-DsRed antibody (magenta) to detect extracellular CD63-mCherry. Scale bar, 10 μm. (I) Boxplot with individual dots representing the relative extracellular CD63-mCherry area in the eye disc for genotypes shown in (F) (*n* = 17, number of clones), (G) (*n* = 21), and (H) (*n* = 17). Thick line, median; ∗∗∗*p* < 0.001; Wilcoxon rank-sum test. (J–K’’’) Eye disc bearing eyFLP-induced MARCM clones of *GFP-Wg/*+ (J), or *UAS-Eiger*^*KB*^, *GFP-Wg/*+ (K) cells stained with anti-GFP antibody (white). Scale bar, 20 μm. (L) Boxplot with individual dots representing the relative GFP-Wg area per clone area of the eye disc for genotypes shown in (J) (*n* = 11, number of clones), and (K) (*n* = 9). Thick line, median; ∗∗∗, *p* < 0.001; Wilcoxon rank-sum test. (M–O) Eye disc bearing eyFLP-induced MARCM clones of wild-type (M), *UAS-Eiger*^*KB*^ (N), or *UAS-Eiger*^*KB*^, *wg/+* (O) cells. Scale bar, 100 μm. (P) Boxplot with individual dots representing the clone size (% of total clone area per eye disc area) in genotypes shown in (M) (*n* = 12, number of eye discs), (N) (*n* = 11), and (O) (*n* = 9). Thick line, median; ∗∗∗*p* < 0.001, ∗∗*p* < 0.01; Wilcoxon rank-sum test.

Although dying cells were observed in Eiger-overexpressing clones generated in the eye discs (Figure S4B, compared to Figure S4A, quantified in Figure S4D), the overall tissue size remained unchanged (quantified in Figure S4E), suggesting that neighboring cells proliferate to maintain tissue homeostasis. We found that the elimination of Eiger-expressing clones and the number of dying cells were significantly suppressed by knocking down *unc-13* in these clones (Figures 4D and S4C, quantified in Figures 4E and S4D), whereas *unc-13-RNAi* expression alone did not affect tissue growth (Figure 4B, compared to Figure 4A, quantified in Figures 4E and S4E). Similarly, in the wing disc, *unc-13* knockdown also significantly suppressed the elimination of Eiger-expressing clones (Figures S4F–S4I, quantified in Figure S4J), suggesting that exocytosis-mediated elimination of JNK-activating cells is not tissue-specific.

In addition, we observed a significant increase in extracellular CD63-mcherry-positive vesicles and GFP-Wg-positive puncta, derived from a knock-in allele,^65^ in Eiger-overexpressing clones in the eye discs, compared to the control (Figures 4F, 4G, 4J’’, and 4K’’, quantified in Figures 4I and 4L). Furthermore, the elimination of Eiger-expressing clones in the eye discs was significantly suppressed to a moderate extent when the Wg signaling gradient was reduced by deleting one copy of the *wg* gene (Figures 4M–4O, quantified in Figure 4P), raising the intriguing possibility that exocytosis-mediated Wg secretion from dying cells may contribute to their cell death and proliferation of neighboring cells.

Interestingly, knocking down *Dronc* in the clones of cells activating JNK signaling by overexpressing the constitutive active form of Hep (Hep^CA^) reduced the increased extracellular CD63-mcherry-positive vesicles (Figures S4K and S4L, quantified in Figure S4M). This suggest that Dronc is crucial for promoting exocytosis downstream of JNK signaling, and that JNK activation alone is not sufficient to maintain exocytic activity in the absence of caspase activation.

Together, these data suggest that JNK signaling promotes exocytosis and the subsequent Wg secretion, which may play a role for maintaining tissue homeostasis. Notably, our findings also highlight a reciprocal interplay between caspase activation and exocytosis, where each process may reinforce the other to sustain apoptotic signaling downstream of JNK signaling.

## Discussion

**Figure 5:**
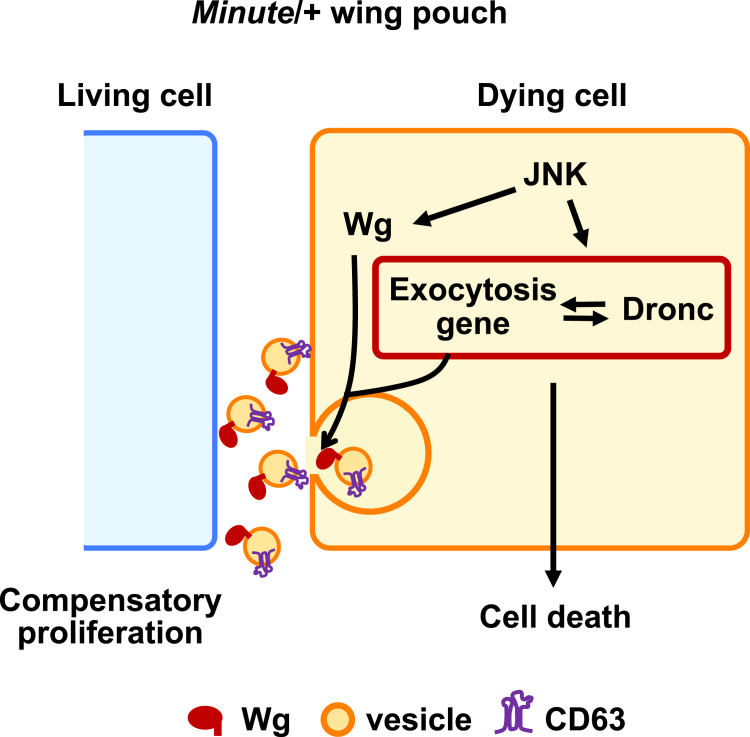
A model for ensuring robust coordination of tissue growth in *M*/+ animals During the larval period of *M*/+ animals, JNK signaling is activated in the *M/+* wing pouch, which induces the expression of exocytosis-related genes and Wg, leading to the secretion of Wg through exocytosis in dying cells. In addition, elevated exocytosis induces cell death via an exocytosis-Dronc amplification loop. This process subsequently promotes the proliferation of neighboring cells, establishing their apoptotic/proliferative status and resulting in significant cell turnover in the *M*/+ wing pouch. Wg (red), vesicles (yellow), and CD63 (purple) are indicated in the schematic.

Our current study reveals that exocytosis is crucial for massive cell turnover in *M/+* wing morphogenesis, acting as a downstream event of JNK signaling. In dying cells, JNK signaling upregulates the expression of exocytosis-related genes and Wg, leading to increased Wg secretion. In parallel, elevated exocytosis, in turn, promotes caspase activation via an exocytosis-Dronc amplification loop, wherein exocytosis enhances Dronc activity, which in turn reinforces exocytosis, driving sustained apoptotic signaling (Figure 5). This exocytosis-mediated Wg secretion and apoptotic feedback loop is a key downstream event of JNK signaling in both *M/+* and Eiger-overexpressing clones, suggesting that exocytosis actively reinforces the apoptotic response, potentially by enhancing JNK signaling to trigger cell death.

Notably, impairment of exocytosis in dying cells in *RpS3/+* animals leads to phenotypic variations in adult wings (Figures 2O and 2P in this study). This highlights the importance of proper exocytosis in maintaining cell turnover and ensuring developmental robustness, which may provide the flexibility needed to adapt developmental programs to varying genetic and environmental conditions.

We identified Wg as an important factor secreted through exocytosis from dying cells, potentially essential for the proliferation of neighboring living cells to maintain tissue homeostasis. Previous studies have shown that “undead” cells, stimulated toward apoptosis but kept alive by the caspase inhibitor p35, are able to promote the proliferation of neighboring cells in the *Drosophila* epithelium.^67^^,^^68^^,^^69^ Several mitogens, including Wg, Dpp, and Spitz (a EGF homolog) have been identified as being produced by apoptotic cells in a JNK-dependent manner.^18^^,^^58^^,^^70^ In addition, Hedgehog (Hh)^15^ have also been identified as products of apoptotic cells. Furthermore, during cell competition between wild-type (winners) and *M/+* cells (losers) in the *Drosophila* epithelium*,* prospective loser cells secrete upd3, which promotes competitive elimination by winner cells.^12^ Hence, in addition to Wg, other mitogens could also be secreted thorough exocytosis from dying cells, potentially contributing to massive cell turnover in the *M/+* wing pouch.

Interestingly, it has been reported that apoptosis triggers a wave of ERK/Akt activity pulses that radially propagate across approximately three healthy cells in a cultured human MCF10A monolayer.^71^ Notably, caspase-activated dying cells have also been shown to induce ERK activation in neighboring cells, which is essential for inhibiting caspase activation and preventing the elimination of clustered cells in the *Drosophila* pupal notum.^72^ Together, these previous studies raise the intriguing possibility that non-autonomous ERK activation and JNK-mediated exocytosis, leading to Wg secretion, may cooperate to maintain tissue homeostasis by modulating the balance between cell death and subsequent cell proliferation. How these pathways interact to orchestrate such a balance remains an open question, requiring further investigation to uncover the precise mechanisms underlying this coordination.

Previous studies have identified exosomes as carriers of Wnt/Wg in the extracellular space of both mammalian and *Drosophila* cells, including wing disc cells.^33^^,^^61^^,^^62^^,^^63^^,^^64^^,^^73^ Concurrently, alternative mechanisms for Wg transport, such as those involving lipoprotein particles or a lipocalin Swim in the *Drosophila* wing disc, have been reported.^32^^,^^74^ Additionally, the cell-surface proteoglycan Dally-like-protein (Dlp) has been reported to enable long-range signaling of the palmitoylated Wg.^65^ Intriguingly, Wnt/Wg transport has also been observed through filopodia-like cellular extensions known as cytonemes.^75^^,^^76^^,^^77^^,^^78^ In our study of the *M*/+ wing disc, a model characterized by massive cell turnover, we observed partial colocalization of GFP-Wg puncta with extracellular CD63-mCherry-positive vesicles. Our data also suggest that Wg secretion through exocytosis may not uniformly occur among all dying cells within this context. This disparity suggests that while exosomes from dying cells significantly contribute to Wg transport within the *M*/+ wing pouch, other pathways may also be operative.

*Rp* genes are crucial in a broad range of organisms, from yeast to humans, frequently exhibiting haploinsufficient mutant phenotypes. Notably, heterozygous mutations in genes encoding ribosomal proteins or ribosomal biogenesis factors are linked to tissue-specific human diseases known as ribosomopathies.^79^ In patients affected by these conditions, some tissues may preserve normal patterning and functions, potentially by buffering stresses caused by ribosomal mutations. Given the evolutionary conservation of the molecules we identified in *Drosophila*, regulating cell turnover through exocytosis in dying cells may represent evolutionarily conserved strategy to prevent developmental abnormalities in ribosomal protein mutants.

### Limitations of the study

Our genetic data suggest that JNK signaling is required for elevated exocytosis, but sustained exocytic activity depends on caspase activation, making it difficult to fully separate the effects of JNK signaling and apoptosis on exocytosis. While our findings support a positive feedback loop between exocytosis and caspase activity, the molecular basis of this interplay remains unclear. Furthermore, although we show that Wg is secreted from dying cells via exocytosis, how this signal is received by neighboring cells and leads to compensatory proliferation is not fully understood. It also remains unclear how the balance between cell proliferation and cell death is regulated to maintain coordinated tissue growth. Future studies investigating the molecular mechanisms as well as the spatial and temporal dynamics of Wg reception and proliferative responses will be essential for a comprehensive understanding of tissue growth coordination.

## Resource availability

### Lead contact

Further information and requests for resources and reagents should be directed to and will be fulfilled by the lead contact, Shizue Ohsawa (ohsawa.shizue.x5@f.mail.nagoya-u.ac.jp).

### Materials availability

This study did not generate new unique reagents.

### Data and code availability

• All data reported in this paper will be shared by the lead contact upon request. The sequencing data have been deposited at DDBJ: PRJDB20450.
• This paper does not report original code.
• Any additional information required to reanalyze the data reported in this paper is available from the lead contact upon request.

## Acknowledgments

We thank Takefumi Kondo, Keisuke Ikawa, Kiichiro Taniguchi, Rina Nagata, and Yukari Sando for discussions, Susumu Tsutsumi, Nana Watanabe, Mina Hoshino, Tomoko Furukawa, and Sayako Suzuki for technical support, and NGS core facility of the Graduate Schools of Biostudies, Kyoto University for supporting the RNA-seq analysis. We also thank Yash Hiromi, Gary Struhl, Maoto Sato, Jean-Paul Vincent, Sharad Kumar, Konrad Basler, the Bloomington *Drosophila* Stock Center (Indiana), the Vienna *Drosophila* Resource Center (Vienna), the National Institute of Genetics Stock Center (Mishima), and the *Drosophila* Genomics and Genetic Resources (Kyoto) for fly stocks. Cell sorting using 10.13039/100017412BD FACS Aria II were performed at the Medical Research Support Center, 10.13039/100022395Graduate School of Medicine, 10.13039/501100005683Kyoto University, supported by Basis for Supporting Innovative Drug 10.13039/100002806Discovery and Life Science Research (BINDS) from 10.13039/100009619AMED (grant no. JP22ama121034). This work was supported in part by 10.13039/501100001700MEXT grant-in-aid (10.13039/501100001691KAKENHI) for Transformative Research Area (A) (grant nos. 20H05945 to S.O., 21H05284 to T.I.), the Scientific Research (B) (grant no. 22H02616 to S.O.), and Challenging Exploratory Research (grant no. 21K19257 and 24K21967 to S.O.), 10.13039/501100002241Japan Science and Technology Agency (10.13039/501100020963Moonshot Research & Development: Grant Number JPMJPS2022 to SO), AMED-10.13039/501100003382CREST, 10.13039/100009619Japan Agency for Medical Research and Development (22gm1710002h0001 to T.I.), 10.13039/100009619Japan Agency for Medical Research and Development (21gm5010001 to T.I.), and 10.13039/100007449Takeda Science Foundation to S.O.

## Author contributions

Conceptualization: N.A., T.I., and S.O. data curation: N.A., T.I., and S.O. formal analysis: N.A. funding acquisition: S.O. and T.I. investigation: N.A., Y.Y., and S.O. methodology: N.A. project administration: S.O. resources: S.O. supervision: S.O. visualization: N.A., T.I., and S.O. writing – original draft: S.O. and N.A. writing – review and editing: N.A., T.I., and S.O.

## Declaration of interests

The authors declare no competing interests.

## STAR★Methods

### Key resources table

**Table:** 

REAGENT or RESOURCE	SOURCE	IDENTIFIER
**Antibodies**		

Rat anti-GFP antibody	Nacalai Tesque	Cat#04404-84; RRID: AB_10013361
Rabbit anti-cleaved PARP (Asp214) antibody	Cell Signaling Technology	Cat#9541; RRID: AB_331426
Rabbit anti-phospho-histone H3 (Ser10) antibody	Cell Signaling Technology	Cat#9706; RRID: AB_331748
Living Colors® dsRed Polyclonal Antibody	Takara Bio	Cat#632496; RRID: AB_10013483
Rabbit anti-GFP antibody	Thermo Fisher Science	Cat#A6455; RRID: AB_221570
Cleaved Drosophila Dcp-1 (Asp216) Antibody	Cell Signaling Technology	Cat#9578; RRID: AB_2721060
mouse anti-β-galactosidase antibody	Sigma-Aldrich	Cat#G8021; RRID: AB_259970
anti-rabbit Alexa 488	Thermo Fisher Science	Cat#A-11034; RRID: AB_2576217
anti-rabbit Alexa 546	Thermo Fisher Science	Cat#A-11035; RRID: AB_2534093
anti-rat Alexa 488	Thermo Fisher Science	Cat#A-11006; RRID: AB_2534074
anti-chicken Alexa 488	Thermo Fisher Science	Cat#A-11039; RRID: AB_2534096
anti-rabbit Alexa 647	Thermo Fisher Science	Cat#A-21245; RRID: AB_2535813
anti-mouse Alexa 647	Thermo Fisher Science	Cat#A-21237; RRID: AB_2535806

**Chemicals, peptides, and recombinant proteins**		

SlowFade Diamond Antifade Mountant	Thermo Fisher Science	Cat#S36972
DAPI	Sigma-Aldrich	Cat#D9542
Glycerol	Sigma-Aldrich	Cat#G5516
n-Propyl Gallate	KANTO CHEMICAL	Cat#32465-31
Schneider’s *Drosophila* Medium	Thermo Fisher Science	Cat#21720024
SYTOX Blue Nucleic Acid Stain	Thermo Fisher Science	Cat#11348
Normal Goat Serum	Jackson ImmunoResearch	Cat#005-000-121; RRID: AB_2336990
Insulin	Sigma-Aldrich	Cat#I0516; CAS: 11070-73-8
10x TrypLE Select	Gibco	Cat#A1217701

**Critical commercial assays**		

NucleoSpin RNA XS Kit	TaKaRa	Cat#740902
NEBNext Ultra II Directional RNA Library Prep Kit for Illumina	NEW ENGLAND Biolabs	Cat#E7760S

**Deposited data**		

RNA-sequencing data	This paper	DDBJ: PRJDB20450

**Experimental models: Organisms/strains**		

*Drosophila melanogaster*: *UAS-EGFP.CD63*	Bloomington Drosophila Stock Center	BDSC91390
*Drosophila melanogaster*: *UAS-CD63.mCherry*	Bloomington Drosophila Stock Center	BDSC91389
*Drosophila melanogaster*: *UAS-Syt1-eGFP*	Bloomington Drosophila Stock Center	BDSC6925
*Drosophila melanogaster*: *20XUAS-IVS-GCaMP6m*	Bloomington Drosophila Stock Center	BDSC42750
*Drosophila melanogaster*: *RpS3*^*Plac92*^	Bloomington Drosophila Stock Center	BDSC5627
*Drosophila melanogaster*: *RpL19*^*K03704*^	Kyoto Stock Center (DGRC)	DGRC102285
*Drosophila melanogaster*: *UAS-unc-13-RNAi*	Bloomington Drosophila Stock Center	VDRC33609
*Drosophila melanogaster*: *UAS-unc-13-RNAi*	National Institute of Genetics	NIG2999R-2
*Drosophila melanogaster*: *UAS-LexA-RNAi*	Bloomington Drosophila Stock Center	BDSC67947
*Drosophila melanogaster*: *UAS-Rab3-RNAi*	National Institute of Genetics	NIG7576R-3
*Drosophila melanogaster*: *UAS-Rab3-RNAi*	Bloomington Drosophila Stock Center	BDSC31691
*Drosophila melanogaster*: *UAS-SNAP25-RNAi*	National Institute of Genetics	NIG HMS01367
*Drosophila melanogaster*: *UAS-SNAP25-RNAi*	Bloomington Drosophila Stock Center	BDSC27306
*Drosophila melanogaster*: *UAS-Syt1-RNAi*	National Institute of Genetics	NIG3139R-1
*Drosophila melanogaster*: *UAS-Syt1-RNAi*	Bloomington Drosophila Stock Center	BDSC31668
*Drosophila melanogaster*: *UAS-Rab27-RNAi*	National Institute of Genetics	NIG14791R-2
*Drosophila melanogaster*: *UAS-Rab27-RNAi*	Bloomington Drosophila Stock Center	BDSC31887
*Drosophila melanogaster*: *UAS-ALiX-RNAi*	National Institute of Genetics	NIG HMS00298
*Drosophila melanogaster*: *UAS-ALiX-RNAi*	Vienna Drosophila Resource Center	VDRC32049
*Drosophila melanogaster*: *Gaq*^*221C*^	Bloomington Drosophila Stock Center	BDSC30744
*Drosophila melanogaster*: *UAS-Gaq-RNAi*	National Institute of Genetics	NIG17759R-2
*Drosophila melanogaster*: *UAS-Gaq-RNAi*	National Institute of Genetics	NIG HMJ30300
*Drosophila melanogaster*: *UAS-Gaq-RNAi*	Bloomington Drosophila Stock Center	BDSC JF02390
*Drosophila melanogaster*: *UAS-Plc21C-RNAi*	National Institute of Genetics	NIG4574R-2
*Drosophila melanogaster*: *UAS-Plc21C-RNAi*	National Institute of Genetics	NIG4574R-3
*Drosophila melanogaster*: *norpA*^*7*^	Kyoto Stock Center (DGRC)	DGRC108362
*Drosophila melanogaster*: *UAS-norpA-RNAi*	Vienna Drosophila Resource Center	VDRC105676
*Drosophila melanogaster*: *UAS-norpA-RNAi*	Bloomington Drosophila Stock Center	BDSC JF02390
*Drosophila melanogaster*: *UAS-Puc*	Martín-Blanco et al.^80^	N/A
*Drosophila melanogaster*: *wg*^*l−*^*^8^*	Bloomington Drosophila Stock Center	BDSC5351
*Drosophila melanogaster*: *UAS-yellow-RNAi*	National Institute of Genetics	NIG3757R-1
*Drosophila melanogaster*: *UAS-Dronc*^*DN*^	Quinn et al.^81^	N/A
*Drosophila melanogaster*: *UAS-Dronc-RNAi*	Vienna Drosophila Resource Center	VDRC23033
*Drosophila melanogaster*: *GFP-Wingless*^*S239A*^	McGough et al.^65^	N/A
*Drosophila melanogaster*: *UAS-VHH4-CD8-HA*	McGough et al.^65^	N/A
*Drosophila melanogaster*: *UAS-Eiger*^*+W*^	Igaki et al.^82^	N/A
*Drosophila melanogaster*: *UAS-Eiger*^*KB*^	Moreno et al.^83^	N/A
*Drosophila melanogaster*: *UAS-Hep*^*CA*^	Bloomington Drosophila Stock Center	BDSC 58781
*Drosophila melanogaster*: *wg*^*026578*^	Bloomington Drosophila Stock Center	BDSC 11205

**Software and algorithms**		

Leica LAS AF Lite software	Leica microsystems	https://leica-las-af-lite.software.informer.com/4.0/
Excel	Microsoft	https://www.microsoft.com/en-gb/microsoft-365/excel
Fiji software	Schindelin et al.^84^	RRID: SCR_002285
RStudio (version 1.2.5033)	RStudio, Inc.	https://rstudio.com
KaleidaGraph	Synergy Software	https://www.synergy.com/

**Other**		

Leica TCS SPE Microscope	Leica Microsystems	https://www.leica-microsystems.com/
Leica STELLARIS 5 Microscope	Leica Microsystems	https://www.leica-microsystems.com/
Leica M205C stereo microscope	Leica Microsystems	https://www.leica-microsystems.com/
ZEISS LSM 900 Microscope		https://www.zeiss.com/microscopy/en/home.html?vaURL=www.zeiss.com/microscopy
Olympus FV3000 Microscope	Olympus Life Science	https://www.olympus-lifescience.com/ja/

### Experimental model and study participant details

#### Fly strains

*Drosophila melanogaster* fly stocks were cultured at 25°C on standard fly food. Larval experiments were conducted with individuals of both sexes, whereas experiments involving adult wings were performed using only females. All experiments were conducted in accordance with the guidelines of Nagoya University and were approved by the Institutional Animal Care and Use Committee (protocol number: 560).

To detect dying cells in the wing pouch, the following strains were used: *nub-Gal4; UAS-CD8-PARP-Venus* (control); *nub-Gal4; UAS-CD8-PARP-Venus, RpS3*^*Plac92*^ (BL5627)*/TM6B* (*RpS3*/+ tester). Additional strains were used as follows: *UAS-EGFP.CD63* (BL91390), *UAS-CD63.mCherry* (BL91389), *UAS-Syt1-eGFP* (BL6925), *RpL19*^*K03704*^ (DGRC102285), *20XUAS-IVS-GCaMP6m* (BL42750), *UAS-unc-13-RNAi* (VDRC33609, NIG2999R-2), *UAS-LexA-RNAi* (BL67947), *UAS-Rab3-RNAi* (NIG7576R-3, BL31691), *UAS-SNAP25-RNAi* (HMS01367, BL27306), *UAS-Syt1-RNAi* (NIG3139R-1, BL31668), *UAS-Rab27-RNAi* (NIG14791R-2, BL31887), *UAS-ALiX-RNAi* (HMS00298, VDRC32049), *UAS-Gαq-RNAi* (NIG17759R-2, HMJ30300, JF02390), *Gαq*^*221C*^ (BL30744), *UAS-Plc21C-RNAi* (NIG4574R-2, 4574R-3), *UAS-norpA-RNAi* (VDRC105676, JF01585), *norpA**^7^* (DGRC108362)*UAS-y-RNAi* (NIG3757R-1), *wg*^*l−8*^ (BL5351), *wg*^*026578*^ (BL11205), *UAS-Puc*,^80^ *UAS-Dronc*^*DN*^(Sharad Kumar),^81^ *UAS-Dronc-RNAi* (VDRC23033), *GFP-Wingless*^*S239A*^ (JP. Vincent),^65^ *UAS-VHH4-CD8-HA* (JP. Vincent),^65^ *UAS-Eiger*^*+W*^,^82^ *UAS-Eiger*^*KB*^ (Konrad Basler),^83^ and *UAS-Hep*^*CA*^ (BL58781).

### Method details

#### Histology

Wandering third-instar larvae were dissected in phosphate buffered saline (PBS) and fixed with 4%-Paraformaldehyde (PFA) for 5 min on ice, followed by 20 min at room temperature. The fixed larvae were washed 3 times with PBT (PBS +0.1% Triton X-100) for 20 min each, and were then blocked with PBTn (5% goat serum (Jackson ImmunoResearch, #005-000-121) + PBT) solution for 30 min. For immunostaining, they were incubated overnight at 4°C with primary antibodies in PBTn. After four 30-min washes with PBT, the samples were re-blocked in PBTn for 30 min, then incubated with secondary antibodies for 2 h at room temperature. Following another set of four 30-min washes with PBT, the samples were mounted using either SlowFade Diamond Antifade Mountant (Invitrogen, #S36972) or an alternative anti-fade mounting medium. The latter was composed of 70% Glycerol (Sigma, #G5516), 0.2% n-Propyl Gallate (KANTO CHEMICAL, #32465-31), and supplemented with DAPI (Sigma, #D9542). For extracellular immunostaining, larvae were dissected in ice-cold Schneider’s *Drosophila* Medium (Gibco, #21720024) and incubated with primary antibodies for 2 h on ice. The larvae were then rinsed with ice-cold Schneider’s *Drosophila* Medium followed by PBS and fixed with 4% PFA for 40 min at room temperature. After fixation, samples were washed with PBS. Subsequent processing was the same as in the preceding protocol. Images were taken with Leica SP5, Leica SP8, Leica SPE, Leica STELLARIS and ZEISS LSM900 confocal microscopes. Primary antibodies used are as follows; rat anti-GFP antibody (Nacalai Tesque, #04404-26, 1:1000) (Extracellular GFP, 1:300), rabbit anti-GFP antibody (Invitrogen, #A6455) (Extracellular GFP, 1:75), rabbit anti-cleaved PARP (Asp214) antibody (Cell Signaling Technology (CST), #9541, 1:200), rabbit anti-phospho-histone H3 (Ser10) antibody (CST, #9706, 1:100), mouse anti-β-galactosidase antibody (Sigma, #G8021, 1:500), rabbit anti-DsRed antibody (Takara Bio Clontech (CLN), #632496, 1:250) (Extracellular dsRed, 1:75), and rabbit anti-Cleaved *Drosophila* Dcp-1 (Asp216) antibody (CST, #9678, 1:100) for 3 days. Secondary antibodies used are as follows; Goat anti-rabbit Alexa 488 (1:250, Invitrogen #A11034), Goat anti-rabbit Alexa 546 (1:250, Invitrogen #A11035), Goat anti-rabbit Alexa 647 (1:250, Invitrogen #A21245), Goat anti-rat Alexa 488 (1:250, Invitrogen #A11006), Goat anti-chicken Alexa 488 (1:250, Invitrogen, #A11039), and Goat anti-mouse Alexa 647 (1:250, Invitrogen #A21237) antibodies.

#### Quantification of puncta number

The vesicle marker EGFP-CD63, CD63-mCherry, and Syt1-eGFP were expressed in the wing pouch under the control of *nub-gal4 driver*. In the region where massive cell death is observed in the *M/+* wing disc, these markers, in addition to GFP-Wingless signals, were acquired at the confocal plane exhibiting the highest signal intensity. CD63-mCherry were also expressed within the mosaic clones of the eye-antenna disc. Puncta were manually counted using Fiji software^84^ and the data were analyzed with KaleidaGraph (Synergy Software).

#### Quantitative analysis of GFP-Wingless and CD63-mCherry colocalization

To analyze the overlap between GFP-Wingless-positive puncta and CD63-mcherry-positive vesicles, their colocalization was manually quantified in the regions exhibiting massive cell death. This quantification was conducted using the confocal plane that displayed the highest signal intensity. Images were manually thresholded to eliminate background signals, and the areas of overlap were calculated using Fiji software, followed by further analyses with KaleidaGraph.

#### Calcium time-lapse imaging and quantification

To monitor Ca^2+^ activity, wandering third-instar larvae were dissected and cultured in Schneider’s *Drosophila* medium supplemented with 2% FBS, 1% Insulin solution (Sigma-Aldrich, #I0516), 3% fly extract (VDRC, #VFE1.25), and 2% low-melting agarose (Sigma-Aldrich, #A4018)/PBS. Imaging was conducted at 10 s intervals for 20-min with a Leica STELLARIS confocal microscope.^49^^,^^85^ For quantitative analysis, dying cells exhibiting continuous calcium activity were manually counted using Fiji software and the data were analyzed with KaleidaGraph. To monitor dying cells, wing discs were incubated in the above medium with 1μM SYTOX (Invitrogen, #11348) for 10 min before imaging.

#### Detection of dying cells and quantification using CD8-PARP-Venus

To detect dying cells, the activated-caspase-3 indicator CD8-PARP-Venus was expressed in the pouch under the control of *nub-gal4* driver. Cleaved PARP signals were acquired at the confocal plane where signal intensity was highest. The number of these signals in the wing pouch was manually measured using Fiji software and the data were analyzed with KaleidaGraph.

#### Quantification of pH3-positive area

For the analysis of pH3-positive cells, total area of pH3-positive cells and the size of the wing pouch area were automatically measured from confocal slices of z stack images compressed using maximum projection functions in Fiji software, as previously described.^86^ The acquired data were then analyzed with KaleidaGraph.

#### Measurement of adult wings

Left and right wings of female flies were rinsed with xylene and mounted in a Canada Balsam (Nacalai Tesque). Images of the wings were acquired using Leica M205C stereo microscope.

#### Quantification of clone size

For the analysis of clone size in the eye disc and wing disc, total clone area as a percentage of the disc area (%) was measured from XY confocal images using Fiji software, as previously described.^86^ The acquired data were then analyzed with KaleidaGraph.

#### FACS and mRNA-seq analysis

GFP+ wing pouch cells were isolated using a FACS Aria II cell sorter (BD Bioscience). Total RNA was then extracted for RNA sequencing analysis, following the method previously described.^87^ Briefly, third-instar larvae were dissected in ice-cold PBS. The wing disc cells were dissociated using Trypsin (10x TrypLE Select) (Gibco, #A1217701) at 37°C for 20 min. The enzymatic reaction was stopped with Schneider’s medium containing 5% FBS, and GFP-positive cells from wing pouches were sorted using a FACS Aria II cell sorter (BD Bioscience). RNA samples were extracted using NucleoSpin RNA XS Kit (TaKaRa, #740902). The quality of the extracted total RNA was assessed using an Agilent 2100 Bioanalyzer, and then Strand-specific Libraries for mRNA-seq were prepared using the NEBNext Ultra II Directional RNA Library Prep Kit for Illumina (E7760S). For RNA-seq, NextSeq 500/550 High output Kit v2.5 was utilized on a NextSeq 500 (Illumine), generating single-end reads at a length of 86 bases.

#### RNA-seq data analysis

Each genotype was analyzed across three independent replicates. Each sequencing experiment generated more than 16 million raw reads. These reads were then quality-filtered using Trim_Galore! (v0.6.5) (https://www.bioinformatics.babraham.ac.uk/projects/trim_galore/) with the default setting to remove low-quality reads and adaptor sequences. The filtered reads were mapped to the *Drosophila* melanogaster genome (Ensembl BDGP6.32), obtained from Illumina iGenomes using STAR.^88^ In the *RpS3/+, nub-Gal4, UAS-Puc* experiment, one sample exhibited only 65.69% of reads being uniquely mapped, which was lower compared to other experiments where more than 80% of reads were uniquely mapped in each sample. The number of reads mapped to each gene was quantified using RSEM (v1.3.3).^89^ Normalization was performed with calcNormFactors function in edgeR (v3.30.3).^90^ Significantly differentially expressed genes were identified with an FDR<0.05 using the glmQLFit and glmQLFTest function in edgeR (R version 4.0.1, limma 3.44.3). Furthermore, GO enrichment analysis was performed on the 362 genes regulated by JNK signaling, utilizing the online tool available at “Gene Ontology” (https://geneontology.org).

### Quantification and statistical analysis

Statistical analysis was performed, and boxplot graphs were generated using KaleidaGraph version4.5 (Synergy Software). All experiments shown in the same graph were conducted at the same time. Thick line in the boxplot represents the median. Significance levels are indicated as follows: ∗*p* < 0.05, ∗∗*p* < 0.01, ∗∗∗*p* < 0.001, and n.s.; not significant. The Wilcoxon rank-sum test was used for non-parametric comparisons. Statistical details are included in the Figure legends, and all statistical data are summarized in Table S4.

## Supplemental information

Document S1. Figures S1–S4

Table S1. List of genes identified as differentially expressed by RNA-seq between RpS3/+ and WT, and between RpS3/+, +Puc and WT pouch cells

Table S2. List of genes differentially expressed in RpS3/+ pouch cells, associated with JNK or JAK/STAT targets, Gr64 gustatory receptors, oxidative stress, and DNA repair

Table S3. List of genes identified as differentially expressed by RNA-seq between RpS3/+ and RpS3/+, +Puc pouch cells

Table S4. Summary of statistical analysis

Table S5. Detailed Genotypes shown in Figures, related to Figure 1-4 and S1-S4

Video S1. Calcium Time-lapse imaging of wing discs, related to Figure 1 The calcium reporter GCaMP6m was expressed in wing pouches of wild-type larvae. Wing discs were dissected and subjected to time-lapse imaging at 10-second intervals with a Leica STELLARIS confocal microscope. See *Methods* for details.

Video S2. Calcium Time-lapse imaging of wing discs, related to Figure 1 The calcium reporter GCaMP6m was expressed in wing pouches of *RpS3/+* larvae. Wing discs were dissected and subjected to time-lapse imaging at 10-second intervals with a Leica STELLARIS confocal microscope. See *Methods* for details.

Video S3. Calcium Time-lapse imaging of wing discs, related to Figure 1 The calcium reporter GCaMP6m was expressed in wing pouches of *RpS3/+*, *nub-Gal4, UAS-Puc* larvae. Wing discs were dissected and subjected to time-lapse imaging at 10-second intervals with a Leica STELLARIS confocal microscope. See *Methods* for details.

Video S4. Calcium Time-lapse imaging of wing discs, related to Figure S1 The calcium reporter GCaMP6m was expressed in wing pouches of *RpS3/+* larvae. Wing discs were dissected, incubated in Schneider’s medium with 1μM SYTOX, and subjected to time-lapse imaging at 10-second intervals with a Leica STELLARIS confocal microscope. See *Methods* for details.

Video S5. SYTOX Time-lapse imaging of wing discs, related to Figure S1 Wing discs from *RpS3/+* larvae were cultured in Schneider’s medium containing 1mM SYTOX, and subjected to time-lapse imaging at 10-second intervals using a Leica STELLARIS confocal microscope. See *Methods* for details.
