# Supplementary material for: JNK signaling coordinates epithelial cell turnover through exocytosis in Drosophila ribosomal protein mutants
Source: iScience. 2025 May 5;28(6):112587. doi: 10.1016/j.isci.2025.112587 (PMC12159887; doi:10.1016/j.isci.2025.112587)
Supplement: Document S1. Figures S1–S4 [file mmc1.pdf]

iScience, Volume *28*

Supplemental information

JNK signaling coordinates epithelial cell turnover through exocytosis in *Drosophila* ribosomal protein mutants

Nanami Akai, Yoshimasa Yagi, Tatsushi Igaki, and Shizue Ohsawa

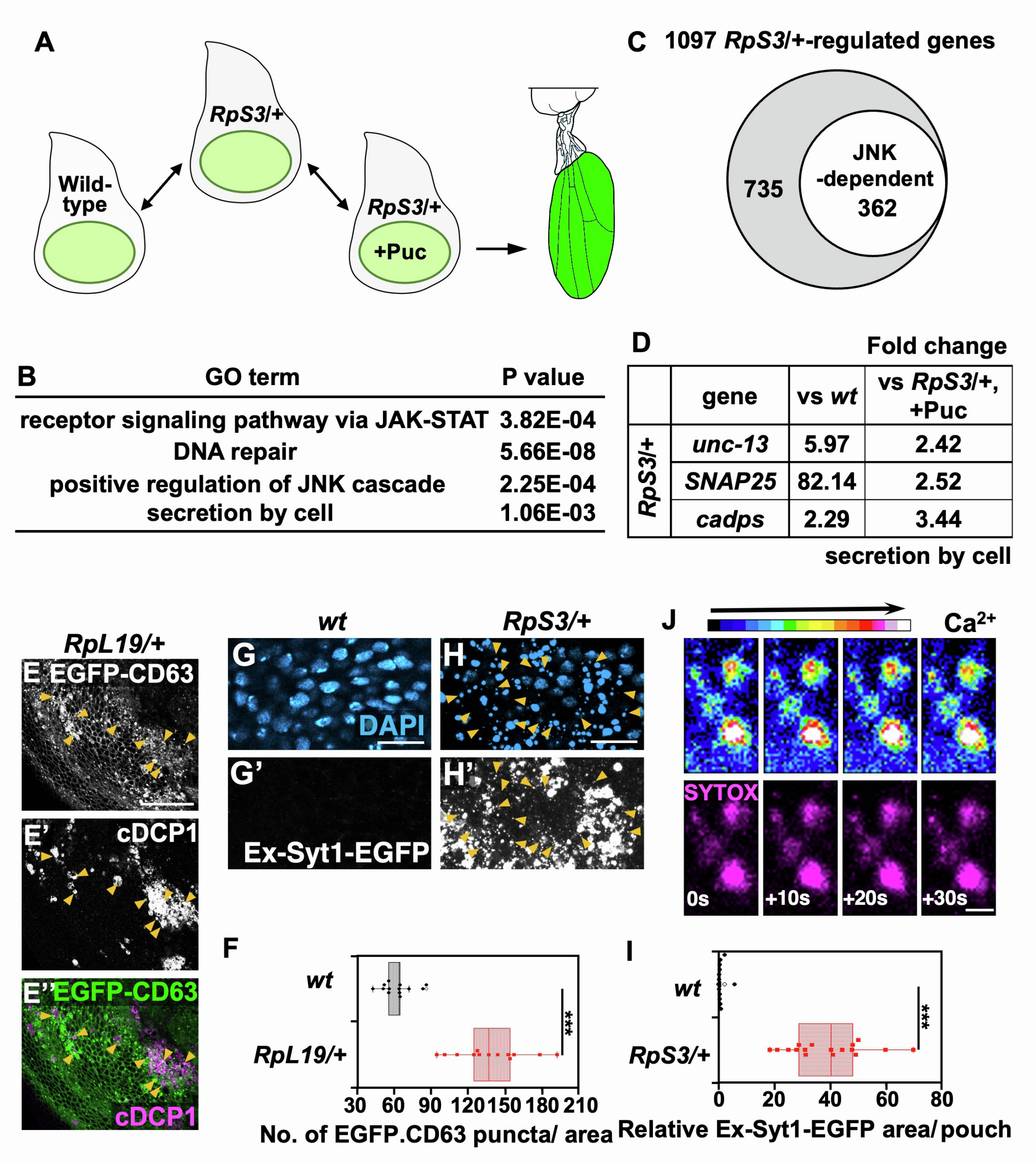
A
Wild-type
RpS3/+
RpS3/+
+Puc
C 1097 RpS3/+-regulated genes
735
JNK
-dependent
362
B
GO term
P value
receptor signaling pathway via JAK-STAT 3.82E-04
DNA repair 5.66E-08
positive regulation of JNK cascade 2.25E-04
secretion by cell 1.06E-03
D
Fold change
gene
vs wt
vs RpS3/+, +Puc
RpS3/+
unc-13 5.97 2.42
SNAP25 82.14 2.52
cadps 2.29 3.44
secretion by cell
RpL19/+
E EGFP-CD63
E' cDCP1
E'' EGFP-CD63
cDCP1
wt
RpS3/+
G
DAPI
H
G'
Ex-Syt1-EGFP
H'
J
Ca2+
SYTOX
0s
+10s
+20s
+30s
F
wt
RpL19/+
***
30 60 90 120 150 180 210
No. of EGFP.CD63 puncta/ area
I
wt
RpS3/+
***
0 20 40 60 80
Relative Ex-Syt1-EGFP area/ pouch

Figure S1. ***M/+* wing pouch elevates exocytosis downstream of JNK signaling.**

Related to Figure 1.

(A) Schematic representation of the genotypes used for transcriptional profiling: mRNA was isolated from FACS-Sorted GFP$^+$ pouch cells (green) in third instar wing discs of wild-type, *RpS3*/+, or *RpS3*/+, *nub-Gal4, UAS-Puc* flies.

(B) Four GO terms for biological process were significantly enriched in the *RpS3/*+-regulated genes.

(C) Venn diagram depicting genes that are differentially expressed in *RpS3*/+ cells compared to wild-type cells, with 362 of these changes being regulated by JNK signaling.

(D) Expression levels (fold changes relative to wild-type control or *RpS3*/+, *nub-Gal4, UAS-Puc*) for *RpS3*/+ -regulated genes associated with the "secretion by cell" GO term. These genes were regulated by JNK signaling in the *RpS3/*+ wing pouch.

(E) EGFP-CD63 was expressed in the wing pouch of *RpL19/*+ flies (white). Dying cells were visualized by anti-cleaved Dcp-1 staining in the wing discs (white). Orange arrowheads indicate massive cell death in the *RpL19/*+ wing pouch. Scale bar, 50 μm.

(F) Boxplot with individual dots representing the number of EGFP-CD63-positive puncta in the pouch of genotypes shown in (Figure 1A) (n=13, number of wing pouches), and (E) (n=13). Thick line, median; ***, p<0.001; Wilcoxon rank-sum test.

(G-H') The vesicle marker Syt1-EGFP was expressed in the wing pouch of wild-type (G), or *RpS3/*+ (H) flies. Extracellular Syt1-EGFP were visualized by anti-GFP staining (white). The nuclei were visualized by DAPI staining (blue). Orange arrowheads indicate the extracellular Syt1-EGFP-positive puncta. The images correspond to the area enclosed by the rectangle 1 in the schematic diagram of the wing disc (Figure 3C). Scale bar, 20μm.

(I) Boxplot with individual dots representing the number of extracellular Syt1-EGFP-positive area in the region enclosed by rectangle 1 in the schematic diagram of the wing disc (Figure 3C) pouch of genotypes shown in (G) (n=13, number of wing pouches), and (H) (n=17). Thick line, median; ***, p<0.001; Wilcoxon rank-sum test.

(J) Time-lapse imaging of Ca^{2+} signaling in cultured wing discs. The calcium reporter GCaMP6m (shown in pseudo color) was expressed in the *RpS3/*+ wing pouch. To monitor dying cells, wing discs were incubated with 1μM SYTOX (magenta). Images were acquired at 10-second intervals. Scale bar, 2 μm.

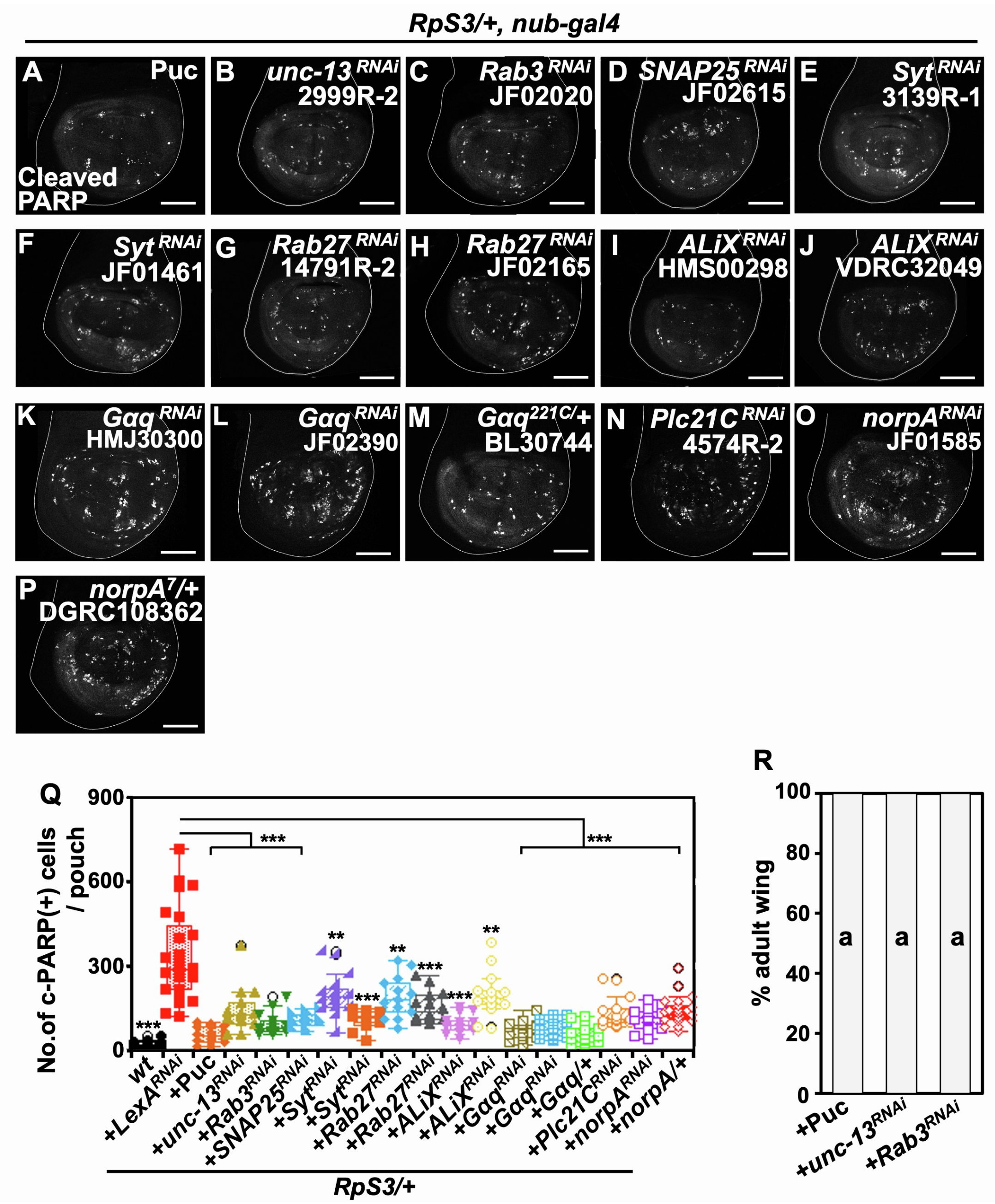

RpS3/+, nub-gal4
A Puc
Cleaved PARP
B unc-13 RNAi 2999R-2
C Rab3 RNAi JF02020
D SNAP25 RNAi JF02615
E Syt RNAi 3139R-1
F Syt RNAi JF01461
G Rab27 RNAi 14791R-2
H Rab27 RNAi JF02165
I ALiX RNAi HMS00298
J ALiX RNAi VDRC32049
K Gαq RNAi HMJ30300
L Gαq RNAi JF02390
M Gαq221C/+ BL30744
N Plc21C RNAi 4574R-2
O norpA RNAi JF01585
P norpA7/+ DGRC108362
Q
No.of c-PARP(+) cells / pouch
900
600
300
0
***
**
wt
+LexA RNAi
+Puc
+unc-13 RNAi
+Rab3 RNAi
+SNAP25 RNAi
+Syt RNAi
+Syt RNAi
+Rab27 RNAi
+Rab27 RNAi
+ALiX RNAi
+ALiX RNAi
+Gαq RNAi
+Gαq RNAi
+Gαq/+
+Plc21C RNAi
+norpA RNAi
+norpA/+
RpS3/+
R
% adult wing
100
80
60
40
20
0
a
+Puc
+unc-13 RNAi
+Rab3 RNAi

Figure S2. Exocytosis is required for massive cell-turnover in the *M/+* wing pouch. Related to Figure 2.

(A-J) CD8-PARP-Venus was expressed in the wing pouch of *RpS3/+, nub-Gal4, UAS-Puc* (A), *RpS3/+, nub-Gal4, UAS-unc-13-RNAi* (B), *RpS3/+, nub-Gal4, UAS-Rab3-RNAi* (C), *RpS3/+, nub-Gal4, UAS-SNAP25-RNAi* (D), *RpS3/+, nub-Gal4, UAS-Syt-RNAi* (E and F), *RpS3/+, nub-Gal4, UAS-Rab27-RNAi* (G and H), *RpS3/+, nub-Gal4, UAS-ALiX-RNAi* (I and J), *RpS3/+, nub-Gal4, UAS-Gaq-RNAi* (K and L), *RpS3/+, Gaq/+* (M), *nub-Gal4, UAS-Plc21C-RNAi* (N), *RpS3/+, nub-Gal4, UAS-norpA-RNAi* (O) or *RpS3/+, norpA/+* (P) flies, and dying cells in the wing pouch were visualized by anti-cleaved PARP staining (white). Scale bar, 100μm.

(Q) Boxplot with individual dots representing the number of cleaved-PARP-positive dying cells per pouch in genotypes shown in (Figure 2G) (n=18, number of wing pouches), (2H) (n=24), (A) (n=18), (B) (n=14), (C) (n=13), (D) (n=12), (E) (n=15), (F) (n=10), (G) (n=13), (H) (n=14), (I) (n=13), (J) (n=13), (K) (n=16), (L) (n=17), (M) (n=15), (N) (n=14), (O) (n=13), and (P) (n=22). Thick line, median; ***, p<0.001, **, p<0.01; Wilcoxon rank-sum test.

(R) The rate of defective wings in the genotypes of *nub-Gal4, UAS-Puc* (n=41), *nub-Gal4, UAS-unc-13-RNAi* (n=55), and *nub-Gal4, UAS-Rab3-RNAi* (n=61).

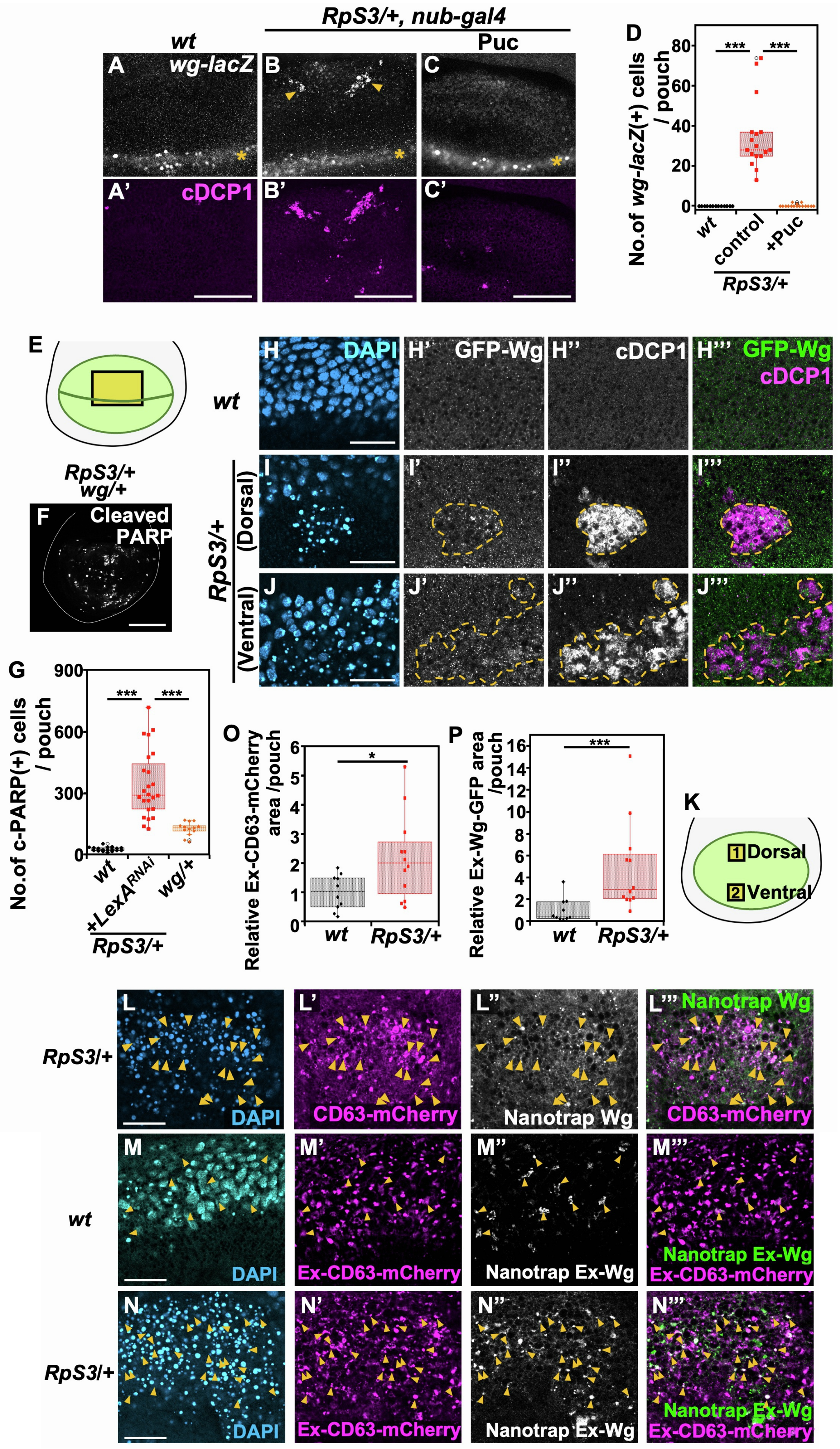
RpS3/+, nub-gal4
wt
Puc
A
wg-lacZ
B
C
A'
cDCP1
B'
C'
D
No.of wg-lacZ(+) cells / pouch
***
***
wt
control
+Puc
RpS3/+
E
RpS3/+
wg/+
F
Cleaved PARP
G
No.of c-PARP(+) cells / pouch
wt
+LexA^{RNAi}
wg/+
RpS3/+
H
DAPI
H'
GFP-Wg
H''
cDCP1
H'''
GFP-Wg
cDCP1
wt
RpS3/+
(Dorsal)
(Ventral)
I
I'
I''
I'''
J
J'
J''
J'''
O
Relative Ex-CD63-mCherry area /pouch
*
wt
RpS3/+
P
Relative Ex-Wg-GFP area /pouch
K
1 Dorsal
2 Ventral
L
L'
L''
L'''
Nanotrap Wg
DAPI
CD63-mCherry
Nanotrap Wg
CD63-mCherry
M
M'
M''
M'''
DAPI
Ex-CD63-mCherry
Nanotrap Ex-Wg
Nanotrap Ex-Wg
Ex-CD63-mCherry
N
N'
N''
N'''
DAPI
Ex-CD63-mCherry
Nanotrap Ex-Wg
Nanotrap Ex-Wg
Ex-CD63-mCherry

Figure S3. Dying cells Secrete Wg via exocytosis in the *M*/+ wing pouch.
Related to Figure 3.

(A-C) Wing discs of *wg-lacZ*/+ (A), *RpS3/+, wg-lacZ/*+ (B), or *RpS3/+, wg-lacZ/+, nub-Gal4, UAS-Puc* (C) flies. Dying cells in the wing pouch were visualized by anti-cleaved Dcp-1 staining (white). *wg* expression was visualized by anti-β-galactosidase staining (white). The endogenous *wg* expression domains are indicated by asterisks (A-C). Orange arrowheads indicate an increase in *wg* expression. The images correspond to the area enclosed by rectangle in the schematic diagram of the wing disc (E). Scale bar, 50μm.

(D) Boxplot with individual dots representing the number of *wg-lacZ*-positive cells per pouch in genotypes shown in (A) (n=13, number of wing pouches), (B) (n=17), and (C) (n=16). Thick line, median; ***, p<0.001; Wilcoxon rank-sum test.

(F) The activated-caspase-3 indicator CD8-PARP-Venus was expressed in the wing pouch of *RpS3/+, wg/*+ flies, and dying cells in the wing pouch were visualized by anti-cleaved PARP staining (white). Scale bar, 100μm.

(G) Boxplot with individual dots representing the number of cleaved-PARP-positive dying cells per pouch in genotypes shown in (Figure 2G) (n=18, number of wing pouches), (Figure 2H) (n=24), and (F) (n=13). Thick line, median; ***, p<0.001; Wilcoxon rank-sum test.

(H-J''') Wing discs from *GFP-Wg*/+ (H), or from the dorsal (I) or ventral (J) regions of the wing disc in *RpS3*/+, *GFP-Wg*/+ flies. The images correspond to the areas enclosed by rectangle 1 and rectangle 2, respectively, in the schematic diagram of the wing disc (K). Dying cells in the wing pouch were stained with anti-cleaved Dcp-1 antibody (white). GFP-Wingless was visualized by anti-GFP staining (white). The regions outlined by dashed lines indicate massive cell death in the *RpS3/*+ wing pouch. Scale bar, 20μm.

(L) CD63-mcherry and Vhh4-CD8-HA were expressed in the wing pouch of *RpS3/*+, *GFP-Wg/*+ flies*.* CD63-mCherry was visualized by anti-DsRed staining (magenta). GFP-Wingless was visualized by anti-GFP staining (white). The nuclei were visualized by DAPI staining (blue). Orange arrowheads indicate the colocalization between CD63-mCherry-positive and GFP-Wingless-positive puncta. The images correspond to the area enclosed by rectangle 1 in the schematic diagram of the wing disc (Figure 3C). Scale bar, 20μm.

(M-N''') Vhh4-CD8-HA was expressed in the wing pouch of *GFP-Wg/*+ (M), or *RpS3/*+, *GFP-Wg/*+ (N) flies. Extracellular CD63-mCherry and extracellular GFP-Wingless were visualized by anti-DsRed staining (magenta) and anti-GFP staining (white), respectively. The nuclei were visualized by DAPI staining (blue). Orange arrowheads indicate the colocalization of extracellular GFP-Wingless-positive puncta with extracellular CD63-mCherry-positive puncta. The images correspond to the area enclosed by the rectangle 1 in the schematic diagram of the wing disc (Figure 3C). Scale bar, 20μm.

(O) Boxplot with individual dots representing extracellular CD63-mCherry-positive area in the region enclosed by rectangle 1 in the schematic diagram of the wing disc (Figure 3C). The respective genotypes are shown in (M) (n=10, number of wing pouches), and (N) (n=12). Thick line, median; *, p<0.05; Wilcoxon rank-sum test.

(P) Boxplot with individual dots representing extracellular GFP-Wingess-positive area in the region enclosed by rectangle 1 in the schematic diagram of the wing disc (Figure 3C). The respective genotypes are shown in (M) (n=10, number of wing pouches), and (N) (n=12) Thick line, median; ***, $p<0.001$; Wilcoxon rank-sum test.

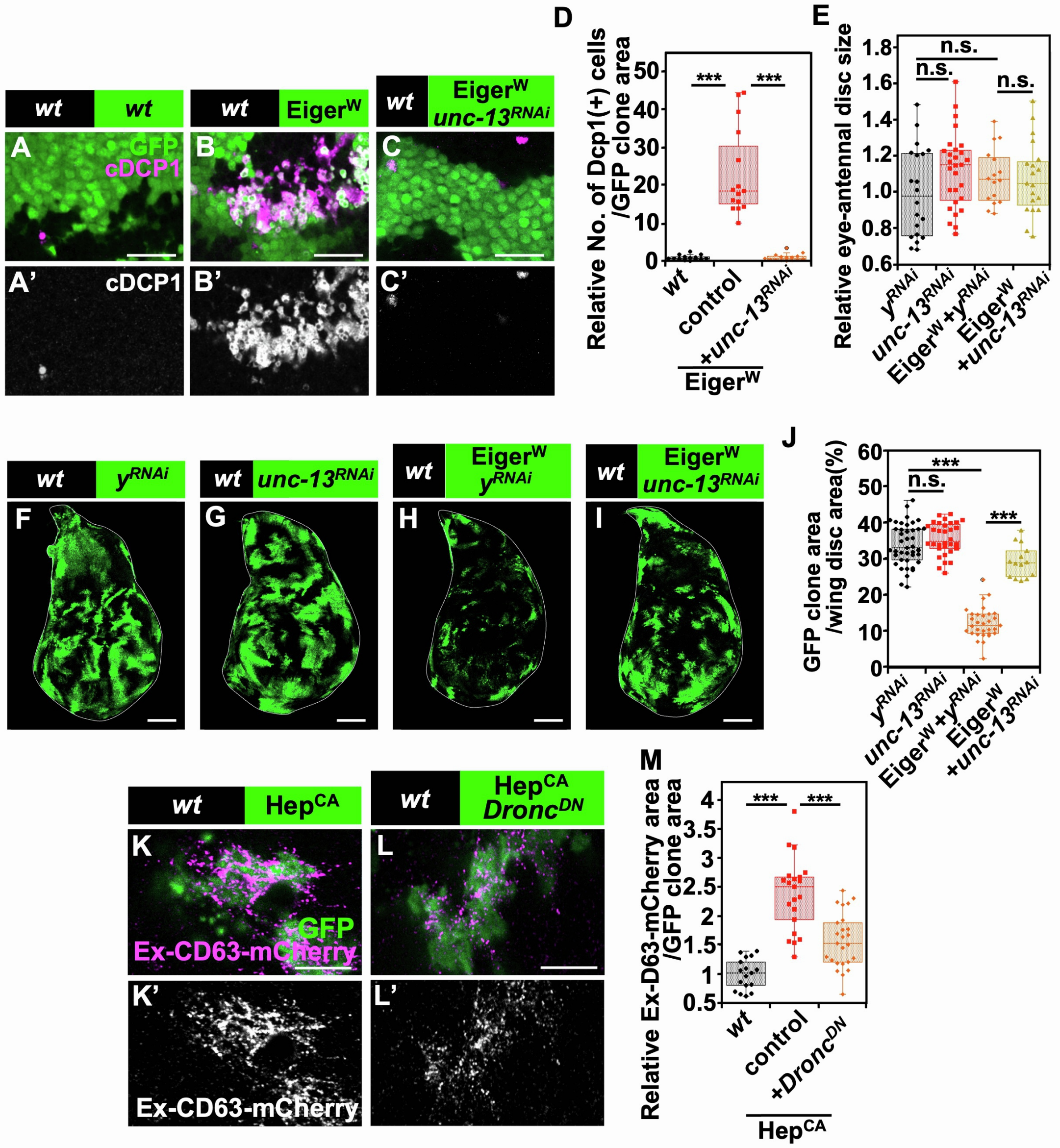

wt
Eiger^W
unc-13^RNAi
GFP
cDCP1
A
B
C
A'
B'
C'
D
E
Relative No. of Dcp1(+) cells /GFP clone area
control
Relative eye-antennal disc size
n.s.
***
F
G
H
I
J
y^RNAi
GFP clone area /wing disc area(%)
K
L
K'
L'
M
Hep^CA
Dronc^DN
Ex-CD63-mCherry
Relative Ex-D63-mCherry area /GFP clone area
+Dronc^DN

Figure S4. JNK signaling universally triggers exocytosis-mediated Wg secretion. Related to Figure 4.

(A-C) Eye disc bearing eyFLP-induced MARCM clones of wild-type (A), *UAS-Eiger*^{W} (B), or *UAS-Eiger*^{W} *+UAS-unc-13-RNAi* (C) cells stained with anti-cleaved Dcp-1. Scale bar, 20μm.

(D) Boxplot with individual dots representing the number of cleaved-Dcp-1-positive dying cells per clone area in genotypes shown in (A) (n=14, number of clones), (B) (n=15), and (C) (n=15). Thick line, median; ***, p<0.001; Wilcoxon rank-sum test.

(E) Boxplot with individual dots representing the eye-antennal disc area in genotypes shown in (Figure 4A) (n=20, number of eye-antennal discs), (Figure 4B) (n=28), (Figure 4C) (n=16), and (Figure 4D) (n=19). Thick line, median; n.s., not significant; Wilcoxon rank-sum test.

(F-I) Wing disc bearing eyFLP-induced MARCM clones of *UAS-yellow-RNAi* (F), *UAS-unc-13-RNAi* (G), *UAS-Eiger*^{W} *+UAS-yellow-RNAi* (H), or *UAS-Eiger*^{W} *+UAS-unc-13-RNAi* (I) cells. Scale bar, 100μm.

(J) Boxplot with individual dots representing the clone size (% of total clone area per wing disc area) in genotypes shown in (F) (n=41, number of wing discs), (G) (n=32), (H) (n=31), and (I) (n=14). Thick line, median; ***, p<0.001; n.s., not significant; Wilcoxon rank-sum test.

(K-L') Eye disc bearing eyFLP-induced MARCM clones of *UAS-Hep*^{CA} *+UAS-CD63-mCherry* (K), or *UAS-Hep*^{CA} *+UAS-Dronc*^{DN} *+UAS-CD63-mCherry* (L) cells, stained with anti-DsRed antibody (magenta) to detect extracellular CD63-mCherry. Scale bar, 10μm.

(M) Boxplot with individual dots representing the relative extracellular CD63-mCherry area in the eye disc for genotypes shown in (Figure 4F, number of clones) (n=17), (K) (n=21), and (L) (n=25). Thick line, median; ***, p<0.001; Wilcoxon rank-sum test.
